# Blood DNA methylation profiles improve breast cancer prediction

**DOI:** 10.1002/1878-0261.13087

**Published:** 2021-11-09

**Authors:** Jacob K. Kresovich, Zongli Xu, Katie M. O’Brien, Min Shi, Clarice R. Weinberg, Dale P. Sandler, Jack A. Taylor

**Affiliations:** ^1^ Epidemiology Branch National Institute of Environmental Health Sciences NIH Research Triangle Park NC USA; ^2^ Biostatistics and Computational Biology Branch National Institute of Environmental Health Sciences NIH Research Triangle Park NC USA; ^3^ Epigenetic and Stem Cell Biology Laboratory National Institute of Environmental Health Sciences NIH Research Triangle Park NC USA

**Keywords:** breast cancer, breast cancer prediction, DNA methylation, risk score

## Abstract

Although blood DNA methylation (DNAm) profiles are reported to be associated with breast cancer incidence, they have not been widely used in breast cancer risk assessment. Among a breast cancer case–cohort of 2774 women (1551 cases) in the Sister Study, we used candidate CpGs and DNAm estimators of physiologic characteristics to derive a methylation‐based breast cancer risk score, mBCRS. Overall, 19 CpGs and five DNAm estimators were selected using elastic net regularization to comprise mBCRS. In a test set, higher mBCRS was positively associated with breast cancer incidence, showing similar strength to the polygenic risk score (PRS) based on 313 single nucleotide polymorphisms (313 SNPs). Area under the curve for breast cancer prediction was 0.60 for self‐reported risk factors (RFs), 0.63 for PRS, and 0.63 for mBCRS. Adding mBCRS to PRS and RFs improved breast cancer prediction from 0.66 to 0.71. mBCRS findings were replicated in a nested case–control study within the EPIC‐Italy cohort. These results suggest that mBCRS, a risk score derived using blood DNAm, can be used to enhance breast cancer prediction.

## Graphical abstract

Blood samples were collected between 2003 and 2009 from a prospective cohort of 50 844 cancer‐free women. Among a case–cohort subsample (2774 women; 1551 incident breast cancers) selected in 2014, DNA methylation was profiled using HumanMethylation450 BeadChips. Elastic net regularization derived a methylation‐based breast cancer risk score (mBCRS). In a validation sample, mBCRS values were higher among cases and improved breast cancer prediction.

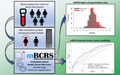

**Abbreviations**
AUC: area under the curve
CI: confidence interval
CpG: cytosine–phosphate–guanine
DCIS: ductal carcinoma *in situ*
DNAm: DNA methylation
EPIC: European Prospective Investigation into Cancer and Nutrition
HR: hazard ratio
mBCRS: methylation‐based breast cancer risk score
OPERA: odds ratio per adjusted standard deviation
OR: odds ratio
PRS: polygenic risk score
SD: standard deviation
SNP: single nucleotide polymorphism

## 1 Introduction

Breast cancer risk is assessed using established risk factors to estimate a woman’s probability of developing the disease [1, 2, 3]. Many breast cancer risk models, including the widely used Breast Cancer Risk Assessment Tool, estimate a woman’s risk using information she provides, such as her age, reproductive history, personal history of benign breast disease, and family history of breast cancer [1]. Genetic models, or polygenic risk scores (PRS), use sets of single nucleotide polymorphisms (SNPs) that are associated with breast cancer to estimate a woman’s germline risk of the disease [3]. Although both questionnaire‐based and genetic breast cancer risk assessment tools appear to be clinically useful [4, 5], incorporating information from the blood epigenome may provide a novel path toward improving breast cancer prediction [6, 7].

Genome‐wide DNA methylation (DNAm) profiles are epigenomic indicators of transcriptional activity, cell function, and identity [8]. DNAm at cytosine–phosphate–guanine (CpG) dinucleotides is associated with breast cancer risk factors, including age, reproductive history, and lifestyle factors [9, 10, 11]. Although DNAm at individual CpG sites is associated with breast cancer risk, such site‐specific associations have been inconsistent across studies [12, 13, 14, 15, 16, 17, 18]. Methylation levels at combinations of many, sometimes hundreds, of CpGs have been shown to be useful in estimating biological age and a variety of other physiologic characteristics [19, 20, 21, 22, 23, 24, 25, 26, 27]. These DNAm estimators are reported to be associated with cancer risk factors [28, 29, 30] and health outcomes, including breast cancer [31, 32, 33], and we hypothesized they could be useful in the development of a methylation‐based risk score for breast cancer.

Using blood samples collected from cancer‐free women, we previously showed that blood DNAm, at individual CpGs and CpG set‐based DNAm estimators, is associated with incidence of breast cancer [17, 18, 31, 32, 33]. Here, after accounting for established questionnaire‐based and genetic risk factors, we examine whether blood DNAm profiles can improve breast cancer prediction. We use a training sample and elastic net regularization [34] to identify a set of individual CpGs and DNAm estimators associated with breast cancer to construct a methylation‐based breast cancer risk score, mBCRS. We validate mBCRS by examining age‐independent associations with breast cancer in a separate testing sample of women from the Sister Study and also in an independent sample of women enrolled in the European Prospective Investigation into Cancer and Nutrition (EPIC) cohort. Finally, we assess the predictive utility of mBCRS alone and in conjunction with questionnaire‐based and genetic risk information.

## 2 Methods

### 2.1 Training and testing set source population, The Sister Study

The Sister Study is a nationwide, ongoing, prospective cohort of 50 884 women residing in the United States and Puerto Rico who were enrolled between 2003 and 2009 [35]. To be eligible, women could not have been diagnosed with breast cancer themselves but must have had a biological sister (full or half) with a previous breast cancer diagnosis. Enrolled women are recontacted annually and are asked to complete short questionnaires about any recent diagnoses, including breast cancer. The annual response rate has consistently been greater than 90%. Women who report an incident breast cancer are contacted six months after diagnosis for permission to retrieve medical records. Written informed consent was obtained at a home visit and the Institutional Review Board of the National Institute of Environmental Health Sciences, National Institutes of Health, approved and oversees the study. The study methodologies conform to the standards set by the Declaration of Helsinki. Data from the Sister Study can be requested via https://sisterstudy.niehs.nih.gov/English/coll‐data.htm.

Blood samples were collected at enrollment (2003–2009) when none of the women had been diagnosed with breast cancer [35]. A case–cohort subsample [36] of non‐Hispanic White women had been selected in July 2014 for whole blood genome‐wide DNAm analysis. As our case set, we identified 1540 participants diagnosed with ductal carcinoma *in situ* (DCIS) or invasive breast cancer during the time between enrollment and the end of February 2014. Approximately 3% (*n* = 1336) of the eligible women from the larger cohort who were cancer‐free at enrollment were randomly selected (the ‘random subcohort’). Of the women selected into the random subcohort, 72 developed incident breast cancer by the end of the study follow‐up period (February 28, 2014).

### 2.2 Genomic DNA methylation data in the Sister Study

Procedures for DNA extraction, processing of Infinium HumanMethylation450 BeadChips, and quality control of DNAm data from Sister Study whole blood samples have been previously described [18]. Of the 2876 women selected for DNAm analysis, 102 samples (61 cases and 41 noncases) were excluded because they did not meet quality control measures. Of these samples, 91 had mean bisulfate intensity less than 4000 or had greater than 5% of probes with low‐quality methylation values (detection *P* > 0.000001, < 3 beads, or values outside three times the interquartile range), four were outliers for their methylation beta value distributions, one had missing phenotype data, and six were from women whose date of diagnosis preceded blood collection [18, 31].

### 2.3 Genomic DNA methylation data in the EPIC‐Italy cohort

DNA methylation raw .idat files (GSE51057) from the EPIC‐Italy nested case–control methylation study [37] were downloaded from the National Center for Biotechnology Information Gene Expression Omnibus website (https://www.ncbi.nlm.nih.gov/geo/). EPIC‐Italy is a prospective cohort with blood samples collected at recruitment; at the time of data deposition, the nested case–control sample included 177 women who had been diagnosed with breast cancer and 152 who were cancer‐free.

### 2.4 DNAm estimator calculation and candidate CpG selection

We used *ENmix* to preprocess methylation data from both studies [38, 39, 40] and applied two approaches to calculate 36 previously established DNAm estimators of biological age and physiologic characteristics (Table S1). We used an online calculator (https://dnamage.genetics.ucla.edu/home) to generate DNAm estimators for eight metrics of epigenetic age acceleration (‘AgeAccel’) [19, 20, 21, 22, 24, 25], telomere length [26], ten measures of white blood cell components [19, 23], and seven plasma proteins (adrenomedullin, β2‐microglobulin, cystatin C, growth differentiation factor‐15, leptin, plasminogen activation inhibitor‐1, and tissue inhibitor metalloproteinase‐1) [25]. We used previously published CpGs and weights to calculate an additional four DNAm estimators for plasma proteins (total cholesterol, high‐density lipoprotein, low‐density lipoprotein, and the total : high‐density lipoprotein ratio) and six complex traits (body mass index, waist‐to‐hip ratio, body fat percent, alcohol consumption, education, and smoking status) [27].

As input to derive the risk score, we also included a set of 100 candidate CpGs previously identified in the Sister Study (Table S2) [18] that were part of the group evaluated in the ESTER cohort study [6] and are available on both the HumanMethylation450 and MethylationEPIC BeadChips.

### 2.5 Statistical analysis

Among women in the Sister Study case‐cohort sample, we randomly selected 70% to comprise a training set; the remaining 30% were used as the testing set for internal validation. Because age is a risk factor for breast cancer, cases were systematically older than noncases at the time of their blood draw. We corrected for this by calculating inverse probability of selection weights. Using the weighted training set, elastic net Cox regression with 10‐fold cross‐validation was applied (using the ‘glmnet’ R package) to identify a subset of DNAm estimators and individual CpGs that predict breast cancer incidence (DCIS and invasive combined). The elastic net alpha parameter was set to 0.5 to balance *L*_1_ (lasso regression) and *L*_2_ (ridge regression) regularization; the lambda penalization parameter was identified using a pathwise coordinate descent algorithm (using the ‘cv.glmnet’ R package) [34]. To generate mBCRS, we created a linear combination of the selected DNAm estimators and CpGs using as weights the coefficients produced by the elastic net Cox regression model.

mBCRS and PRS associations with breast cancer incidence were examined using covariate‐adjusted standardized residuals in the testing set by estimating hazard ratios (HRs) and 95% confidence intervals (CI) and calculating 2‐sided *P*‐values from Cox regression models for case‐cohort designs with Barlow weights, robust standard errors, and age as the timescale [41, 42]. Because age was treated as the timescale, all resulting HRs are fully adjusted for age. Standardized residuals for mBCRS and PRS were calculated by using the data from the random subcohort and regressing each factor separately on a set of established, questionnaire‐based risk factors (i.e., age at blood draw, menopause status, body mass index, physical activity, alcohol consumption, age at first birth [among parous], total number of births, age at menarche, menopause age [among postmenopausal], smoking pack‐years, previous number of breast biopsies, number of breast cancer affected first‐degree family members, youngest age of proband sister’s diagnosis, educational attainment, durations of postmenopausal hormone use, and breastfeeding), and standardizing the residual by dividing it by the standard deviation of the residuals. For our main analysis, our case definition included both DCIS and invasive breast cancers. To explore the possible influence of clinically occult breast cancer, associations were also examined after excluding the first 2 years of follow‐up. In all analyses using the testing set, we excluded women if they were missing information on self‐reported breast cancer risk factors (*n* = 12) or PRS (*n* = 27). Although questionnaire‐based and genetic risk information was not available for the EPIC‐Italy nested case–control study, we calculated standardized residuals for mBCRS adjusted only for age and examined breast cancer associations using odds ratios (ORs) estimated by unconditional logistic regression models.

After accounting for the previously mentioned questionnaire‐based risk factors, we examined risk gradients for mBCRS, PRS, and age, alone and in combination. A risk gradient is defined as the ability to differentiate cases from controls on a population basis and is estimated as odds ratio per adjusted standard deviation (OPERA) using a logistic regression model [43]. In both the Sister Study testing set and the EPIC‐Italy sample, we further examined mBCRS predictive utility using receiver operating characteristic analysis to calculate area under the curve (AUC). To examine whether mBCRS provides additional information for breast cancer prediction, in the Sister Study testing set, we compare AUCs using a sequential combination of the set of previously mentioned questionnaire‐based risk factors, 313 SNP PRS, and mBCRS [44].

## 3 Results

There were 1551 incident breast cancer diagnoses among the 2774 women selected into the methylation case‐cohort sample (Table 1). Overall, the average age at blood draw was 57 years [standard deviation (SD) = 9; range: 35–74). Fewer than 40% of women reported having been tested for *BRCA1* and *BRCA2* and among these women, the self‐reported mutation prevalence was 5% and 6%, respectively. The training set had more invasive cancers (80%) and fewer DCIS (20%) than the testing set (74% invasive, 26% DCIS). After age‐based inverse probability of selection weighting of the training set, there was no difference between cases and noncases by age at blood draw; however, cases had higher weighted mean PRS values, lower levels of physical activity, higher alcohol consumption, older ages at first birth, more affected family members, and a greater proportion with a history of breast biopsy (Table S3).

**Table 1:** Sister Study methylation case‐cohort sample characteristics at study enrollment overall and by training/testing set assignment.

	Overall	Training set	Testing set	*P*‐diff
Total participants, *N*	2774	1941	833	
Incident breast cancers, *N* (%)	1551 (100)	1090 (100)	461 (100)	
Invasive	1218 (79)	877 (80)	341 (74)	0.01
DCIS	333 (21)	213 (20)	120 (26)	
Age, mean years (SD)	57.0 (9)	57.3 (9)	56.5 (9)	0.03
Body mass index, mean kg/m^2^ (SD)	27.6 (6)	27.6 (6)	27.7 (6)	0.53
Physical activity, mean METs/week (SD)	50.8 (31)	50.6 (31)	51.3 (32)	0.60
Alcohol consumption, mean drinks/week (SD)	3.1 (5)	3.1 (5)	3.2 (5)	0.60
Age at menarche, mean years (SD)	12.6 (1)	12.6 (1)	12.6 (2)	0.58
Live births, mean total (SD)	1.9 (1)	2.0 (1)	1.9 (1)	0.13
Age at first birth, mean years (SD)	24.9 (5)	24.9 (5)	24.9 (5)	0.86
Breastfeed duration, mean weeks (SD)	35.2 (56)	35.4 (54)	34.6 (61)	0.73
Postmenopausal hormone use^a^, mean years (SD)	5.9 (7)	5.9 (7)	5.6 (7)	0.98
Number of affected sisters, count (SD)	1.1 (0.4)	1.1 (0.4)	1.1 (0.4)	0.51
Youngest proband sister age at diagnosis, mean years (SD)	48.9 (10)	49.0 (10)	48.6 (10)	0.27
Polygenic risk score, mean (SD)	−0.17 (0.6)	−0.16 (0.7)	−0.19 (0.6)	0.18
Educational attainment, *N* (%)				
Less than HS/HS degree	430 (16)	311 (16)	119 (14)	0.40
Attended college/college degree	1641 (59)	1134 (58)	507 (61)	
Advanced degree	703 (25)	496 (26)	207 (25)	
Menopause status, *N* (%)				
Premenopausal	826 (30)	574 (30)	252 (30)	0.73
Postmenopausal	1947 (70)	1366 (70)	581 (70)	
Previous number of breast biopsies, *N* (%)				
Zero	1875 (68)	1312 (68)	563 (68)	0.25
One	448 (17)	330 (17)	158 (19)	
Two or more	411 (15)	299 (15)	112 (13)	

METs, metabolic equivalent tasks. Missing covariates: body mass index, 2; sister age at diagnosis, 3; polygenic risk score, 102; menopause status, 1; postmenopausal hormone use, 6. *P*‐diff calculated using the *t*‐tests for continuous variables and χ^2^ tests for categorical variables.

^a^ Among postmenopausal women (*n* = 1947).

Among the weighted training set, elastic net regularization selected 5 DNAm estimators and 19 individual CpGs to comprise mBCRS (Table S4). These included two estimators of epigenetic age acceleration (PhenoAgeAccel, Raj AgeAccel) and three white blood cell subtype proportions (CD8+ T cells, monocytes, and CD8+CD28‐CD45RA‐). Of the 19 individual CpGs selected, 12 mapped to genes, including the following: *BTNL9*, *GLTSCR2*, *CYTSB*, *COQ10B*, *LHFP*, *NUMB*, *WWTR1*, *PSMA1*, *SLAIN1*, *XRCC2*, *SPTY2D1*, and *KCTD18*. The components of mBCRS were generally not correlated with each other (Fig. S1). Women diagnosed with breast cancer over follow‐up had higher mBCRS scores based on its original scale (mean difference = 0.13; Fig. S2) or based on the standardized residuals (mean difference = 0.66; Fig. S3).

### 3.1 mBCRS associations with genetic and questionnaire‐based breast cancer risk factors

**Fig. 1:**
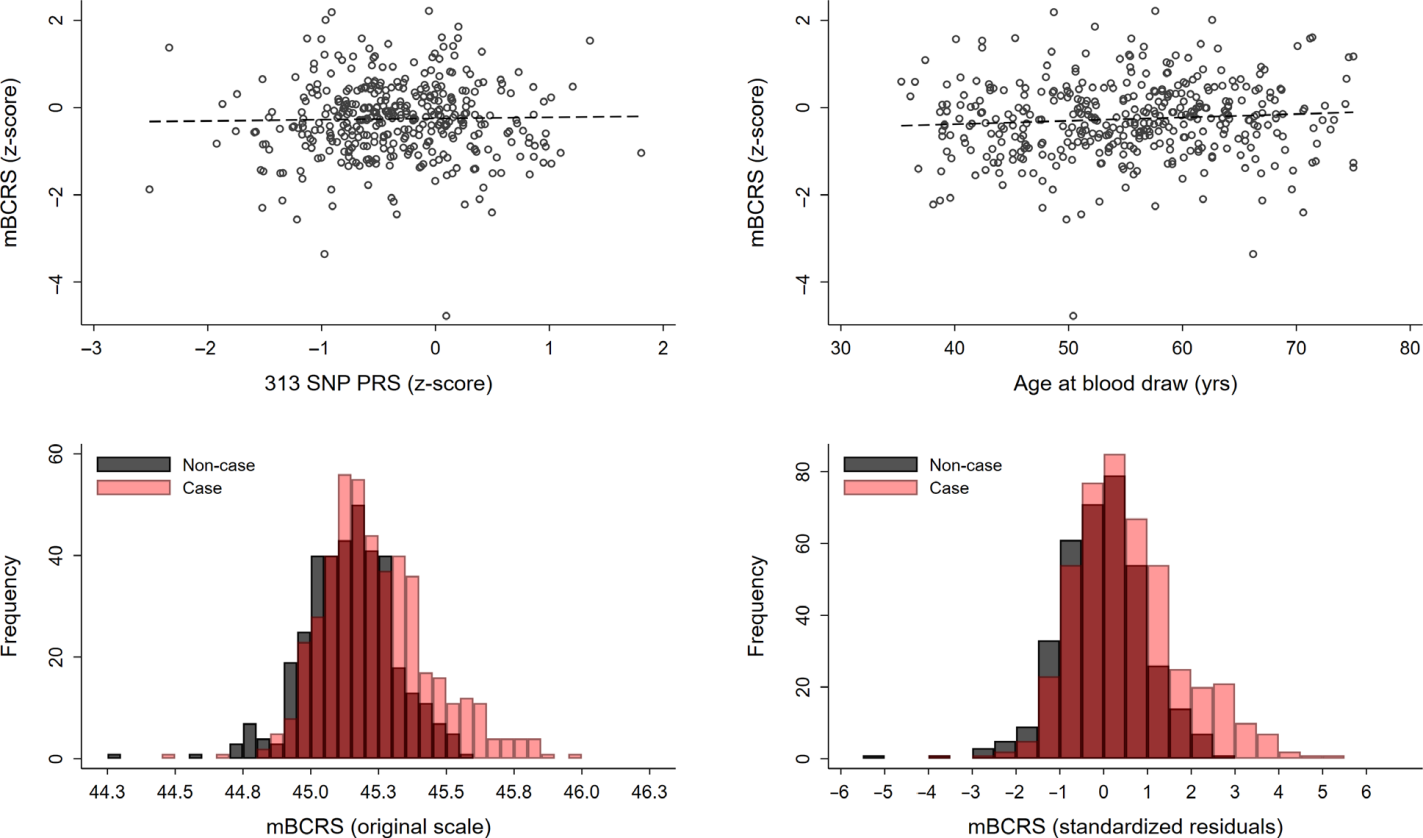
mBCRS correlations with 313 SNP PRS and age and mBCRS distributions in the Sister Study testing set. Among women sampled as part of the random subcohort and selected into the testing set (*n* = 375), scatterplot and fit line between mBCRS values and the 313 SNP PRS (Pearson’s correlation: 0.03, top left panel); and scatterplot and fit line between mBCRS and age at blood draw (Pearson’s correlation: 0.09, top right panel). The distribution of mBCRS on the original scale ranged from 44.28 to 45.96, with a mean of 45.20 (SD = 0.19); women who remained cancer‐free had a mean of 45.14 (SD = 0.17), those who developed breast cancer had a mean of 45.24 (SD = 0.20) (bottom left panel). The standardized residuals for mBCRS ranged from −5.30 to 5.20, with a mean of 0.30 (SD = 1.19); women who remained cancer‐free had a mean of −0.03 (SD = 0.99), and those who developed breast cancer had a mean of 0.56 (SD = 1.27) (bottom right panel).

Among women in the random subcohort who were selected into the Sister Study testing set, mBCRS was not correlated with either the 313 SNP PRS (*r* = 0.03, *P* = 0.51) or age at blood draw (*r* = 0.09, *P* = 0.07) (Fig. 1, top row). The distribution of mBCRS on the original scale ranged from 44.28 to 45.96, with a mean of 45.20 (SD = 0.19); women who remained cancer‐free had a mean of 45.14 (SD = 0.17), while those who developed breast cancer had a mean of 45.24 (SD = 0.20) (Fig. 1, bottom row left). The standardized residuals for mBCRS ranged from −5.30 to 5.20, with a mean of 0.30 (SD = 1.19); women who remained cancer‐free had a mean of −0.03 (SD = 0.99), while those who developed breast cancer had a mean of 0.56 (SD = 1.27) (Fig. 1, bottom row right). Eighteen of the 24 components selected into the mBCRS were uncorrelated with age (*P* > 0.05); the strongest positive correlation was observed for the CD8+CD28‐CD45RA‐ cell type (*r* = 0.29, *P* < 0.001), and the strongest negative correlation was observed for cg02456218 (*r* = −0.19, *P* < 0.001) (Fig. S4). In the EPIC‐Italy sample, among the controls, mBCRS was positively correlated with age (*r* = 0.20, *P* = 0.01). In the random subcohort members of the Sister Study testing set, the PRS and age were not correlated (*r* = 0.01, *P* = 0.88; Fig. S5). mBCRS was not correlated with reproductive factors including the following: age at menarche, age at first live birth, number of births, age at menopause, duration of postmenopausal hormone use, or breastfeeding (all *P* > 0.05; Fig. S6). mBCRS was also not correlated with previous number of breast biopsies, number of affected family members, proband sister age at diagnosis, smoking history, physical activity, alcohol use, or educational attainment (all *P* > 0.05), but was positively correlated with body mass index (*r* = 0.11, *P* = 0.03; Fig. S7).

### 3.2 mBCRS and breast cancer incidence

In the testing set of the Sister Study, higher mBCRS was associated with elevated breast cancer risk (per covariate‐adjusted SD, HR: 1.78, 95% CI: 1.51, 2.11, *P* = 2.1 × 10^−11^), showing similar strength to the 313 SNP PRS (per covariate‐adjusted SD, HR: 1.57, 95% CI: 1.35, 1.83, *P* = 5.1 × 10^−9^; Table 2). In a multivariable model that included standardized residuals for both mBCRS and PRS, associations were unchanged (Table 2). Similar associations between mBCRS and breast cancer risk were observed in the EPIC‐Italy external validation set (per age‐adjusted SD increase, OR: 2.11, 95% CI: 1.62, 2.76, *P* = 4.5 × 10^−8^). In tertile‐based analyses, Sister Study participants with mBCRS scores in the highest third had over a twofold increase in breast cancer risk (Tertile 3 vs Tertile 1, HR: 2.54, 95% CI: 1.78, 3.63, *P* = 2.9 × 10^−7^; *P*‐trend = 3.6 × 10^−7^); in the EPIC‐Italy sample, participants with mBCRS scores in the highest third had an over fourfold increase in risk (Tertile 3 vs Tertile 1, OR: 4.18, 95% CI: 2.33, 7.49, *P* = 1.7 × 10^−6^; *P*‐trend = 1.8 × 10^−6^) (Table S5). In the Sister Study testing set, a LOWESS plot of mBCRS standardized residuals by case status showed some evidence of nonlinearity (Fig. S8). To examine the possibility that the association between mBCRS and breast cancer was secondary to the presence of clinically occult tumors, we excluded the first 2 years of follow‐up: mBCRS again showed positive associations with breast cancer incidence (per covariate‐adjusted SD, HR: 1.83, 95% CI: 1.52, 2.19, *P* = 1.1 × 10^−10^; PRS HR: 1.62, 95% CI: 1.37, 1.92, *P* = 1.3 × 10^−8^); again, associations were similar in a multivariable model (Table 2). Among women who developed breast cancer, mBCRS was weakly correlated with time to diagnosis (*r* = −0.09, *P* = 0.05) (Fig. S9). mBCRS associations with breast cancer incidence did not vary by age at blood draw, body mass index, menopausal status or sister proband age at diagnosis (Table S6). In a case‐only analysis to investigate etiologic heterogeneity, mBCRS was more strongly associated with invasive breast cancer than DCIS (invasive, per covariate‐adjusted SD, HR: 1.90, 95% CI: 1.58, 2.29, *P* = 1.5 × 10^−11^; DCIS, HR: 1.50, 95% CI: 1.20, 1.89, *P* = 4.1 × 10^−4^; etiologic heterogeneity, *P* = 0.05); however, no differences were observed when comparing mBCRS and occurrence of estrogen receptor positive and negative invasive breast cancers (etiologic heterogeneity, *P* = 0.47; Table S7).

**Table 2:** Test set‐based hazard ratios per covariate‐adjusted standard deviation (SD) from univariable and multivariable analyses of mBCRS and polygenic risk score (*n* = 794).

	Full follow‐up^a^			Excluding first 2 years		
	HR (95% CI)^b^	*Z*‐score	*P*‐value	HR (95% CI)^b^	*Z*‐score	*P*‐value
Univariate models^c^						
mBCRS	1.78 (1.51, 2.11)	6.70	2.1 × 10^−11^	1.83 (1.52, 2.19)	6.45	1.1 × 10^−10^
PRS	1.57 (1.35, 1.83)	5.84	5.1 × 10^−9^	1.62 (1.37, 1.92)	5.69	1.3 × 10^−8^
Multivariable model^d^						
mBCRS	1.74 (1.46, 2.06)	6.23	4.6 × 10^−10^	1.76 (1.47, 2.12)	6.03	1.6 × 10^−9^
PRS	1.55 (1.31, 1.83)	5.14	2.8 × 10^−7^	1.59 (1.33, 1.92)	4.98	6.3 × 10^−7^

Sample sizes: full follow‐up, *n* = 794 with 443 events; excluding first 2 years, *n* = 673 with 326 events.

^a^ Full follow‐up length: mean = 5.2 years, SD = 2.6.

^b^ Per covariate‐adjusted standard deviation increase in mBCRS score (or PRS), accounting for: age at blood draw, menopause status, body mass index, interaction term for BMI and menopause, physical activity, alcohol consumption, age at first birth (among parous), total number of births, age at menarche, menopause age (among postmenopausal), smoking pack‐years, previous number of breast biopsies, family history of breast cancer (number of affected sisters, youngest age of sister’s diagnosis), educational attainment, and durations of postmenopausal hormone use and breastfeeding, and standardized to the distribution of the noncases.

^c^ Results displayed are from two separate models including either mBCRS or PRS.

^d^ Results displayed are for both mBCRS and PRS that were included as covariates in a single model.

### 3.3 Breast cancer risk gradients and predictive capabilities of mBCRS, PRS and other risk factors

**Fig. 2:**
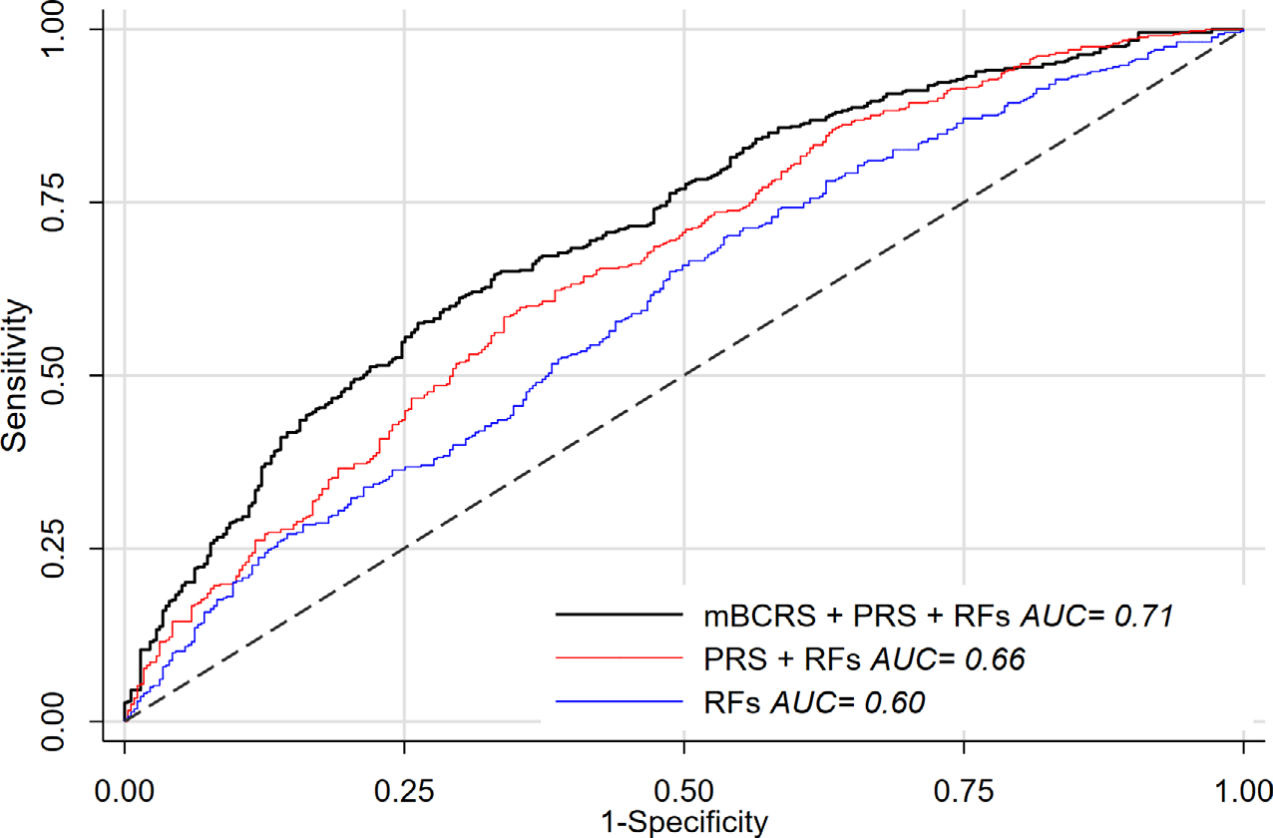
Predictive capability of breast cancer risk markers in Sister Study testing set. Predictive capability of the mBCRS, PRS, and questionnaire‐based breast cancer risk factors (RFs) using receiver operating characteristics analysis; predictive capability reported as AUC. Questionnaire‐based risk factors include the following: age at blood draw, menopause status, body mass index, physical activity, alcohol consumption, age at first birth (among parous), total number of births, age at menarche, menopause age (among postmenopausal), smoking pack‐years, previous number of breast biopsies, family history of breast cancer (number of affected sisters, youngest proband sister age at diagnosis), educational attainment, and durations of postmenopausal hormone use and breastfeeding.

In univariable models that account for questionnaire‐based breast cancer risk factors, the OPERAs for mBCRS, PRS, and age were 1.58 (95% CI: 1.38, 1.81), 1.58 (95% CI: 1.36, 1.83), and 1.35 (95% CI: 1.16, 1.57), respectively (Table 3). Multivariable models with different combinations of mBCRS, PRS, and age produced OPERA estimates similar to those of univariate models (Table 3). Multivariate OPERA estimates for the two classes of mBCRS predictors (i.e., the 5 separate DNAm estimators and the 19 individual CpGs) separately and combined show that components from each of the two classes were independently predictive of breast cancer incidence (Table S8). In the EPIC‐Italy validation set, the AUC for mBCRS was 0.69 (95% CI: 0.63, 0.75). In the Sister Study testing set, the AUC was 0.63 (95% CI: 0.59, 0.67) for mBCRS, 0.63 (95% CI: 0.59, 0.67) for PRS, and 0.60 (95% CI: 0.56, 0.64) for the set of questionnaire‐based risk factors listed in the methods. Combining PRS and questionnaire‐based risk information produced an AUC of 0.66 (95% CI: 0.62, 0.70); when mBCRS was added to that model, the AUC rose to 0.71 (95% CI: 0.67, 0.74, *P*‐diff = 1.7 × 10^−4^) (Fig. 2).

**Table 3:** Test set‐based OPERA (95% CI) estimated odds ratios per covariate‐adjusted standard deviation from univariable and multivariable analyses of mBCRS, polygenic risk score, and age (*n* = 794).

	Univariable			Multivariable			
	mBCRS only	PRS only	Age only	mBCRS + PRS	mBCRS + age	PRS + age	All three combined
mBCRS	1.58 (1.38, 1.81)	–	–	1.58 (1.37, 1.81)	1.56 (1.36, 1.79)	–	1.56 (1.35, 1.79)
Polygenic risk score	–	1.58 (1.36, 1.83)	–	1.57 (1.35, 1.84)	–	1.61 (1.38, 1.87)	1.60 (1.37, 1.87)
Age at blood draw^a^	–	–	1.35 (1.16, 1.57)	–	1.31 (1.12, 1.53)	1.38 (1.19, 1.62)	1.35 (1.15, 1.58)
Log likelihood (null = −545.0)	−520.1	−525.8	−537.4	−502.4	−514.1	−517.1	−495.5
LR χ^2^ DF^b^	1	1	1	2	2	2	3
χ*^2^*	49.9	38.5	15.3	85.3	61.8	55.9	99.0

Breast cancer risk factors include the following: age at blood draw, menopause status, body mass index, interaction term for BMI and menopause, physical activity, alcohol consumption, age at first birth (among parous), total number of births, age at menarche, menopause age (among postmenopausal), smoking pack‐years, previous number of breast biopsies, family history of breast cancer (number of affected sisters, youngest age of sister’s diagnosis), educational attainment, and durations of postmenopausal hormone use and breastfeeding.

^a^ Residuals adjusted for all breast cancer risk factors, except for age at blood draw.

^b^ LR χ^2^ DF, likelihood ratio χ^2^ statistic degrees of freedom.

## 4 Discussion

We used epigenome‐wide DNAm array data from the Sister Study, a large prospective cohort of women, to construct a novel DNAm‐based risk score for breast cancer. Using published methods, we calculated a set of 36 DNAm estimators of biological age and physiologic characteristics and also included a candidate set of 100 individual CpGs previously reported to be associated with breast cancer [18]. Elastic net regularization was used with a training set of women in the Sister Study to identify 5 DNAm estimators and 19 CpGs that together jointly predicted breast cancer incidence. In a separate testing set of women from the Sister Study, the resulting metric, mBCRS, was strongly associated with breast cancer risk; the association was also verified in an independent study of women from the EPIC cohort. OPERA analysis supported the conclusion that mBCRS, PRS, and age are complementary and independent predictors of breast cancer risk. Both OPERA estimates and AUCs calculated by receiver operating characteristic analysis suggest that breast cancer prediction based on genetic and questionnaire‐based information can be meaningfully improved with the addition of mBCRS.

Like the individual CpGs, most of the DNAm estimators selected for mBCRS have been reported as markers of breast cancer risk. PhenoAgeAccel was associated with breast cancer incidence in both the Sister Study and EPIC cohorts [24, 33], and DNAm estimators of circulating CD8+ T cells and monocytes appear to be time‐dependent markers of breast cancer risk [31]. Although not previously reported, our analysis suggested the Raj AgeAccel metric and DNAm estimator for CD8+CD28‐CD45RA‐ immune cells may also be risk markers for breast cancer.

Established breast cancer risk factors include the PRS, which is based on 313 SNPs associated with the disease [3], and questionnaire‐based risk factors including age, body mass index, alcohol use, reproductive factors, history of benign breast disease, and family history of breast cancer [45, 46, 47, 48, 49]. In our analysis, mBCRS was not correlated with PRS, age, or most other breast cancer risk factors. Unlike some breast cancer risk factors [50], we did not find evidence that mBCRS associations varied by degree of family history or other personal characteristics consistent with the possibility that the risk associated with mBCRS acts multiplicatively with other breast cancer risk factors. We did observe some evidence that the association between mBCRS and breast cancer risk may be nonlinear, with the strongest associations among women with the highest scores. In order to assess whether mBCRS improved breast cancer prediction, we used receiver operating characteristic curves to examine change in AUC based on different sets of risk factors. Change in AUC is dependent on the order in which variables are entered; using a conservative approach of first including questionnaire‐based and genetic information, we found that breast cancer prediction was markedly improved with the subsequent inclusion of mBCRS, another indication that blood DNA methylation provides new information related to breast cancer risk. We also examined this question using OPERA estimates, which presumes a logit‐linear relationship between the covariate‐adjusted standardized residual and the outcome. Although our data suggest nonlinearity, the OPERAs for mBCRS were remarkably consistent across univariable and multivariable analyses. Perhaps most importantly, in the OPERA analysis the estimates for mBCRS were similar to those of PRS and those reported for new mammogram‐based measures [51, 52, 53], placing it among the strongest known risk factors for breast cancer [54].

Our study is not without limitations. All women enrolled in the Sister Study cohort had a biological sister previously diagnosed with breast cancer, and they are therefore at higher risk of breast cancer than the general population [35]. Although the rapid accrual rate provided by this design improves the ability to identify environmental, epigenetic, and genetic risk factors for breast cancer [55], the study of women at higher risk of disease may limit the generalizability of our findings. However, we validated mBCRS in the independent EPIC‐Italy cohort, a study that was not restricted to women with a family history of breast cancer. Another potential limitation is that we used for model input a candidate set of individual CpGs that were previously reported to be associated with breast cancer risk in the Sister Study. However, we applied 10‐fold cross‐validation to protect against overfitting, and again the validation in EPIC data is supportive. Our sample was restricted to non‐Hispanic White women; mBCRS associations with breast cancer risk in other race/ethnicities have yet to be explored. Like genotype information used in the PRS, epigenome‐wide DNAm data are more expensive to obtain than the self‐reported risk factor information. While genotyping costs have decreased rapidly and genotypes are now available for large numbers of individuals, the lower availability of methylation array data in large prospective studies of breast cancer currently limits wider investigation. Presumably, these costs will also come down, particularly if blood DNAm profiles are found to be clinically useful. Finally, although the inclusion of additional breast cancer risk factors such as mammographic‐based measures may provide further improvements [51, 52, 53, 54], the level of risk discrimination may remain modest, as in our models that combine available questionnaire‐based risk information, PRS and mBCRS.

## 5 Conclusions

mBCRS, a novel risk score derived using blood DNAm array data, predicts breast cancer incidence. mBCRS captures risk that is distinct from both genetic and questionnaire‐based information and is similar in magnitude to that captured by the 313 SNP PRS. The addition of a methylation‐based risk score for breast cancer, mBCRS, to existing genetic and questionnaire‐based information resulted in markedly improved breast cancer prediction.

## Conflict of interest

The authors declare no conflict of interest.

## Author contributions

JKK and JAT conceived the study idea, drafted, and edited the manuscript. JKK performed the data analysis. ZX and MS helped with data management. KMO, CRW, and DPS provided constructive edits to the manuscript. All authors approved of the final version of the manuscript.

### Peer Review

The peer review history for this article is available at https://publons.com/publon/10.1002/1878‐0261.13087.

## Supporting information


**Fig. S1.** Correlation matrix for individual mBCRS components.
**Fig. S2.** Histogram depicting the distribution of mBCRS values in the training set by case status.
**Fig. S3.** Histogram depicting the distribution of the standardized residuals for mBCRS in the training set by case status.
**Fig. S4.** mBCRS component correlations with age.
**Fig. S5.** Correlation between age and PRS values.
**Fig. S6.** mBCRS correlations with selected reproductive factors.
**Fig. S7.** mBCRS correlations with selected breast cancer risk factors.
**Fig. S8.** LOWESS plot of mBCRS by case status.
**Fig. S9.** Correlation between mBCRS and time to diagnosis among cases.
**Table S1.** List of 36 DNAm estimators of biological age and physiologic characteristics included as input to derive the methylation‐based breast cancer risk score.
**Table S2.** List of 100 CpGs associated with breast cancer risk identified by Xu *et al*. (2019) and included as input to derive the methylation‐based breast cancer risk score.
**Table S3.** Characteristics of the training set, weighted to account for differences in age at blood draw.
**Table S4.** DNAm‐based components and coefficients selected to comprise mBCRS.
**Table S5.** mBCRS tertile associations with breast cancer incidence in the Sister Study internal validation set and the EPIC‐Italy external validation set.
**Table S6.** mBCRS associations (per covariate‐adjusted SD) with breast cancer incidence in the Sister Study testing set, stratified by age at blood draw, body mass index, menopause status and proband age at diagnosis.
**Table S7.** mBCRS associations (per covariate‐adjusted SD) with breast cancer incidence by invasiveness and estrogen receptor status, and tests for etiologic heterogeneity in the Sister Study testing set.
**Table S8.** Multivariate OPERA (95% CI) estimates of odds ratios per adjusted standard deviation from separate and combined analysis of mBCRS component classes (DNAm estimators & individual CpGs), adjusted for breast cancer risk factors and standardized.

## Data Availability

Data from the Sister Study can be requested via https://sisterstudy.niehs.nih.gov/English/coll‐data.htm.
