# Supplementary material for: Blood DNA methylation profiles improve breast cancer prediction
Source: Mol Oncol. 2021 Nov 9;16(1):42–53. doi: 10.1002/1878-0261.13087 (PMC8732352; doi:10.1002/1878-0261.13087)
Supplement: Supplementary file 1 — Fig. S1. Correlation matrix for individual mBCRS components. Fig. S2. Histogram depicting the distribution of mBCRS values in the training set by case status. Fig. S3. Histogram depicting the distribution of the standardized residuals for mBCRS in the training set by case status. Fig. S4. mBCRS component correlations with age. Fig. S5. Correlation between age and PRS values. Fig. S6. mBCRS correlations with selected reproductive factors. Fig. S7. mBCRS correlations with selected breast cancer risk factors. Fig. S8. LOWESS plot of mBCRS by case status. Fig. S9. Correlation between mBCRS and time to diagnosis among cases. Table S1. List of 36 DNAm estimators of biological age and physiologic characteristics included as input to derive the methylation‐based breast cancer risk score. Table S2. List of 100 CpGs associated with breast cancer risk identified by Xu et al. (2019) and included as input to derive the methylation‐based breast cancer risk score. Table S3. Characteristics of the training set, weighted to account for differences in age at blood draw. Table S4. DNAm‐based components and coefficients selected to comprise mBCRS. Table S5. mBCRS tertile associations with breast cancer incidence in the Sister Study internal validation set and the EPIC‐Italy external validation set. Table S6. mBCRS associations (per covariate‐adjusted SD) with breast cancer incidence in the Sister Study testing set, stratified by age at blood draw, body mass index, menopause status and proband age at diagnosis. Table S7. mBCRS associations (per covariate‐adjusted SD) with breast cancer incidence by invasiveness and estrogen receptor status, and tests for etiologic heterogeneity in the Sister Study testing set. Table S8. Multivariate OPERA (95% CI) estimates of odds ratios per adjusted standard deviation from separate and combined analysis of mBCRS component classes (DNAm estimators & individual CpGs), adjusted for breast cancer risk factors and standardized. [file MOL2-16-42-s001.docx]

| Supplemental Table 1. List of 36 DNAm estimators of biological age and physiologic characteristics included as input to derive the methylation-based breast cancer risk score | | |
| --- | --- | --- |
| Estimator | Output from online calculator | Reference |
| *Epigenetic Age Acceleration* | | |
| Horvath AgeAccel | Yes | (1) |
| Hannum AgeAccel | Yes | (2) |
| PhenoAgeAccel | Yes | (3) |
| RajAgeAccel^1^ | Yes | (4) |
| GrimAgeAccel | Yes | (5) |
| IEAA | Yes | (6) |
| EEAA | Yes | (6) |
| IEAA Hannum | Yes | (2) |
| *Telomere length* |  |  |
| Telomere length | Yes | (7) |
| *White Blood Cell Components* | | |
| CD8+ T-cells | Yes | (8) |
| CD4+ T-cells | Yes | (8) |
| Natural Killer | Yes | (8) |
| B-cell | Yes | (8) |
| Monocytes | Yes | (8) |
| Granulocytes | Yes | (8) |
| Plasmablast | Yes | (1) |
| CD8+CD28-CD45RA- | Yes | (1) |
| CD8+ naïve T-cells | Yes | (1) |
| CD4+ naïve T-cells | Yes | (1) |
| *Plasma Proteins* |  |  |
| Adrenomedullin | Yes | (5) |
| β2-microglobulin | Yes | (5) |
| Cystatin C | Yes | (5) |
| Growth differentiation factor-15 | Yes | (5) |
| Leptin | Yes | (5) |
| Plasminogen activation inhibitor 1 | Yes | (5) |
| Tissue inhibitor metalloproteinase 1 | Yes | (5) |
| Total cholesterol | No | (9) |
| High density lipoprotein | No | (9) |
| Low density lipoprotein | No | (9) |
| Total:HDL ratio | No | (9) |
| *Complex Traits* | | |
| Body mass index | No | (9) |
| Waist-to-hip ratio | No | (9) |
| Body fat % | No | (9) |
| Alcohol consumption | No | (9) |
| Education | No | (9) |
| Smoking status | No | (9) |
| ^1^Also known as “skin & blood” epigenetic age acceleration metric  Abbreviations: intrinsic epigenetic age acceleration, IEAA; extrinsic epigenetic age acceleration, EEAA.  Online calculator: https://dnamage.genetics.ucla.edu/home | | |

| Supplemental Table 2. List of 100 CpGs associated with breast cancer risk identified by Xu et al. (2019) (10) and included as input to derive the methylation-based breast cancer risk score | | | |
| --- | --- | --- | --- |
| IlmnID | Chromosome | Position | Gene |
| cg00162673 | chr1 | 51700514 | RNF11 |
| cg11750112 | chr1 | 64938742 | CACHD1 |
| cg02083528 | chr1 | 78280850 | FAM73A |
| cg09664975 | chr1 | 84543551 | PRKACB |
| cg21584883 | chr1 | 93645960 | TMED5;CCDC18 |
| cg09136695 | chr1 | 111722281 | CEPT1 |
| cg06698332 | chr1 | 151967449 | S100A10 |
| cg16008440 | chr1 | 161983790 | OLFML2B |
| cg25768273 | chr1 | 211911579 |  |
| cg07031542 | chr1 | 214567074 | PTPN14 |
| cg13865248 | chr1 | 214842875 |  |
| cg00008800 | chr2 | 38710171 |  |
| cg03754165 | chr2 | 60780427 | BCL11A |
| cg05999729 | chr2 | 98369433 |  |
| cg24654185 | chr2 | 99812274 | MRPL30 |
| cg23562675 | chr2 | 120107745 | C2orf76 |
| cg01580574 | chr2 | 134949671 |  |
| cg05059169 | chr2 | 198339611 | COQ10B |
| cg22902939 | chr2 | 201355148 | KCTD18 |
| cg03555299 | chr2 | 206830681 |  |
| cg02540338 | chr2 | 217243695 |  |
| cg21331107 | chr3 | 32541698 | CMTM6 |
| cg01551787 | chr3 | 72982378 | GXYLT2 |
| cg19385799 | chr3 | 78164711 |  |
| cg10723575 | chr3 | 149422266 | WWTR1 |
| cg23535170 | chr3 | 179489892 | USP13 |
| cg01021224 | chr5 | 35049428 | AGXT2 |
| cg01885839 | chr5 | 111017556 |  |
| cg10825350 | chr5 | 132541078 | FSTL4 |
| cg19780570 | chr5 | 133764548 |  |
| cg02456218 | chr5 | 180476573 | BTNL9 |
| cg23819836 | chr6 | 15663134 | DTNBP1 |
| cg19082069 | chr6 | 26018534 | HIST1H1A |
| cg06079966 | chr6 | 26237621 |  |
| cg24152718 | chr6 | 30185818 |  |
| cg19494811 | chr6 | 34356253 | NUDT3 |
| cg07439409 | chr6 | 97731296 | MIR548H3;C6orf167 |
| cg05099288 | chr6 | 109700555 | CD164 |
| cg17345859 | chr7 | 6728525 | ZNF12 |
| cg24495177 | chr7 | 64362326 | ZNF273 |
| cg10412943 | chr7 | 97739696 | LMTK2 |
| cg06193393 | chr7 | 99869454 | GATS |
| cg21921829 | chr7 | 116850153 | ST7 |
| cg06479434 | chr7 | 128395929 | CALU |
| cg20864326 | chr7 | 152371422 | XRCC2 |
| cg22277672 | chr7 | 158444325 | NCAPG2 |
| cg17477493 | chr8 | 12748272 |  |
| cg12927915 | chr8 | 66542625 | ARMC1 |
| cg13155823 | chr8 | 80993721 | TPD52 |
| cg12861602 | chr8 | 99045811 | MATN2 |
| cg01296653 | chr9 | 4678949 | CDC37L1 |
| cg21246531 | chr9 | 36039639 | RECK |
| cg14569423 | chr9 | 126523663 | DENND1A |
| cg16719077 | chr10 | 7849111 | ATP5C1 |
| cg25248628 | chr10 | 22518027 |  |
| cg23260111 | chr10 | 46158095 | ANUBL1 |
| cg01433914 | chr10 | 111792543 | ADD3 |
| cg08320413 | chr10 | 127455161 | MMP21 |
| cg03539267 | chr10 | 133823899 |  |
| cg13544125 | chr11 | 14661502 | PSMA1 |
| cg21107103 | chr11 | 18630052 | SPTY2D1 |
| cg20006187 | chr11 | 44410456 |  |
| cg20667822 | chr11 | 48153832 | PTPRJ |
| cg14908986 | chr11 | 70120720 | PPFIA1 |
| cg22152887 | chr11 | 72849944 | FCHSD2 |
| cg22473961 | chr12 | 56119258 | CD63 |
| cg13425650 | chr12 | 94093761 | CRADD |
| cg25282652 | chr12 | 128364739 |  |
| cg11496226 | chr12 | 129571803 | TMEM132D |
| cg05099186 | chr13 | 39923838 | LHFP |
| cg25399333 | chr13 | 50856824 |  |
| cg04139515 | chr13 | 78272639 | SLAIN1 |
| cg16574347 | chr13 | 78327370 | SLAIN1 |
| cg18462916 | chr13 | 113918539 | CUL4A |
| cg13979573 | chr14 | 21584590 |  |
| cg11720863 | chr14 | 24604040 | PSME1 |
| cg23173466 | chr14 | 65565498 | MAX |
| cg05180258 | chr14 | 73921198 | NUMB |
| cg22731164 | chr15 | 40192158 | GPR176 |
| cg25217313 | chr15 | 41624774 | NUSAP1;OIP5 |
| cg13402936 | chr15 | 50471108 |  |
| cg04474209 | chr15 | 52484920 | MYO5C;GNB5 |
| cg04258046 | chr15 | 63445591 | RPS27L |
| cg16743903 | chr16 | 89593216 | SPG7 |
| cg02704946 | chr17 | 6527184 | KIAA0753 |
| cg01691987 | chr17 | 7789028 | CHD3 |
| cg03117577 | chr17 | 20187457 | CYTSB |
| cg27657459 | chr17 | 54853771 |  |
| cg09628499 | chr17 | 56299729 |  |
| cg04256065 | chr17 | 76102509 | TNRC6C |
| cg16954236 | chr18 | 8638786 | RAB12 |
| cg23419170 | chr18 | 12067067 |  |
| cg12361352 | chr18 | 31158540 | ASXL3 |
| cg05692899 | chr18 | 77342368 |  |
| cg18569070 | chr19 | 17136562 | CPAMD8 |
| cg10812466 | chr19 | 32718114 |  |
| cg06632759 | chr19 | 37702089 | ZNF585B |
| cg02679291 | chr19 | 48248902 | GLTSCR2 |
| cg25325053 | chr20 | 58533022 | CDH26 |
| cg01162841 | chrX | 19818905 | SH3KBP1 |

| Supplemental Table 3. Characteristics of the training set, weighted to account for differences in age at blood draw (n= 1,866) | | | |
| --- | --- | --- | --- |
|  | Non-cases | Cases | *P-diff* |
| Total participants, N | 809 | 1,057 |  |
| Age, mean yrs. (SD) | 57.5 (8) | 57.5 (9) | 0.97 |
| Polygenic risk score, mean (SD) | -0.1 (0.7) | -0.3 (0.6) | < 0.01 |
| Body mass index, mean kg/m^2^ (SD) | 27.4 (5) | 27.7 (6) | 0.36 |
| Physical activity, mean METs/wk. (SD) | 52.2 (30) | 49.3 (32) | 0.05 |
| Alcohol consumption, mean drinks/wk. (SD) | 2.8 (4) | 3.3 (5) | 0.05 |
| Age at menarche, mean yrs. (SD) | 12.6 (1) | 12.6 (1) | 0.85 |
| Live births, mean total (SD) | 2.0 (1) | 1.9 (1) | 0.23 |
| Age at first birth, mean yrs. (SD) | 24.5 (5) | 25.3 (6) | < 0.01 |
| Breastfeed duration, mean wks. (SD) | 36.9 (52) | 34.8 (57) | 0.40 |
| Postmenopausal hormone use^1^, mean yrs. (SD) | 5.6 (6) | 6.1 (8) | 0.19 |
| Number of affected family members, count (SD) | 1.1 (0.3) | 1.2 (0.5) | 0.03 |
| Youngest proband sister age at diagnosis, yrs. mean (SD) | 49.2 (9) | 49.0 (10) | 0.73 |
| Educational attainment, (%) |  |  | 0.10 |
| Less than HS/HS degree | 17.4 | 14.6 |  |
| Attended college/college degree | 58.9 | 58.3 |  |
| Advanced degree | 23.6 | 27.2 |  |
| Menopause status, (%) |  |  | 0.44 |
| Premenopausal | 26.8 | 28.5 |  |
| Postmenopausal | 73.2 | 71.5 |  |
| Previous number of breast biopsies, (%) |  |  | < 0.01 |
| Zero | 71.1 | 64.4 |  |
| One | 16.4 | 17.8 |  |
| Two or more | 12.6 | 17.8 |  |
| Note: n= 1,866 (75 women, including 34 cases and 41 non-cases, were missing information on PRS values  ^1^Among postmenopausal women  Abbreviations: ductal carcinoma in situ, DCIS; metabolic equivalent tasks, METs. | | | |

| Supplemental Table 4. DNAm-based components and coefficients selected to comprise mBCRS | |
| --- | --- |
| *DNAm estimators (5)* | *Coefficient* |
| PhenoAgeAccel | 0.002573167 |
| Raj AgeAccel | 0.009841455 |
| CD8+ T-cells | -0.744294199 |
| Monocytes | -0.283641885 |
| CD8+CD28-CD45RA- | -0.003969645 |
| *Individual CpGs (19)* |  |
| cg00008800 | -1.041803691 |
| cg01885839 | -6.913917889 |
| cg02456218 | -1.114136078 |
| cg02679291 | 0.059842651 |
| cg03117577 | 2.689463151 |
| cg03555299 | -2.157284869 |
| cg05059169 | -0.752195971 |
| cg05099186 | 0.642067947 |
| cg05180258 | -0.390757824 |
| cg09628499 | -2.107281397 |
| cg10723575 | -2.360593857 |
| cg10812466 | -0.607899668 |
| cg13544125 | 1.608556542 |
| cg16574347 | -1.747282628 |
| cg17477493 | -0.750147542 |
| cg19385799 | -4.571092125 |
| cg20864326 | -1.454530303 |
| cg21107103 | -0.534006491 |
| cg22902939 | 66.593775615 |
| CpGs mapped to genes: cg02456218, *BTNL9*; cg02679291, *GLTSCR2*; cg03117577, *CYTSB*; cg05059169, *COQ10B*; cg05099186, *LHFP*; cg05180258, *NUMB*; cg10723575, *WWTR1*; cg13544125, *PSMA1*; cg16574347, *SLAIN1*; cg20864326, *XRCC2*; cg21107103*,* *SPTY2D1*; cg22902939, *KCTD18*. | |


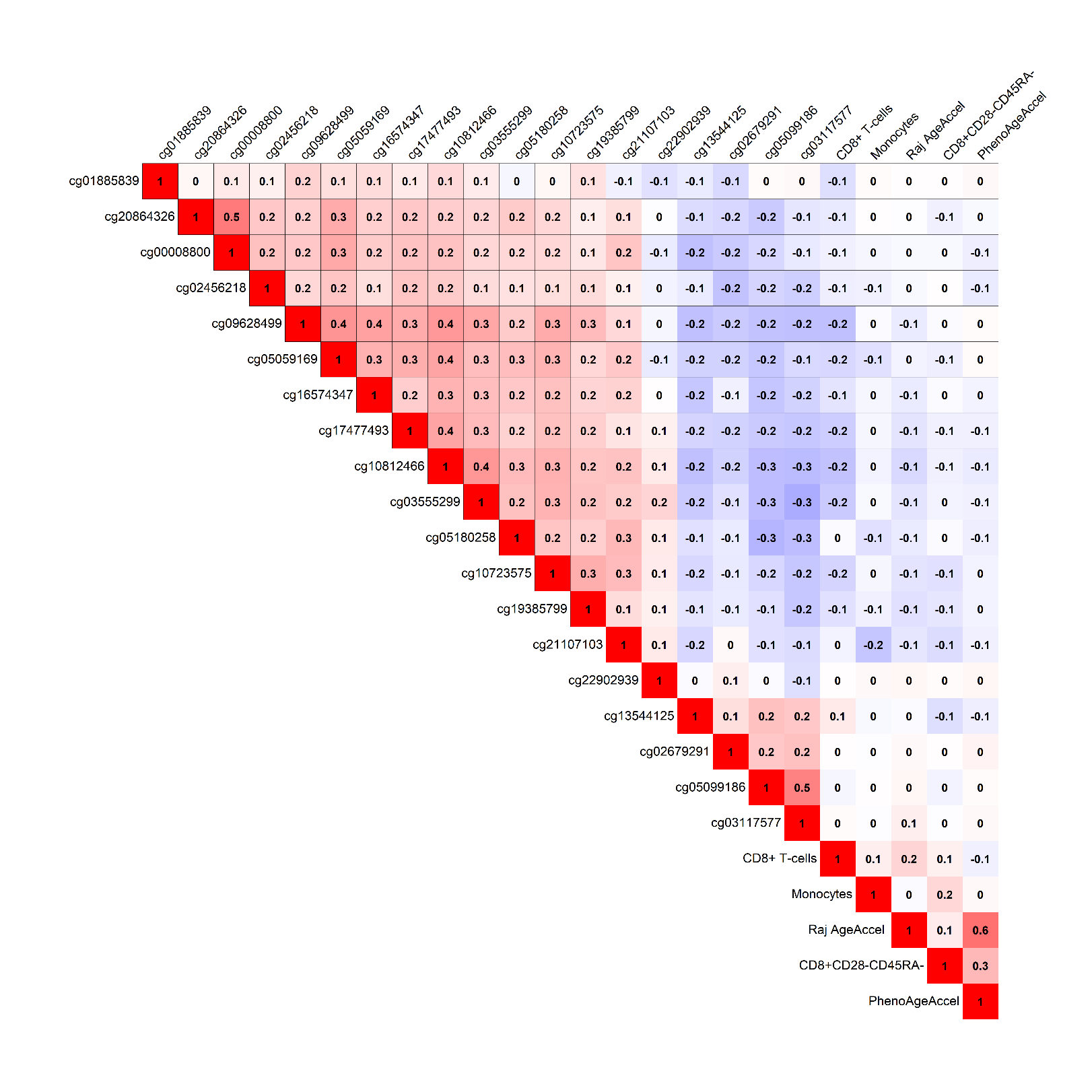


Supplemental Figure 1. Correlation matrix for individual mBCRS components. Among women sampled as part of the random subcohort and selected in the training set (n= 899), Pearson correlation matrix for the individual DNAm components selected to comprise mBCRS. In total, 5 DNAm estimators and 19 individual CpGs were selected into the mBCRS. Pearson correlations between the individual components were modest, ranging between -0.3 to 0.6.


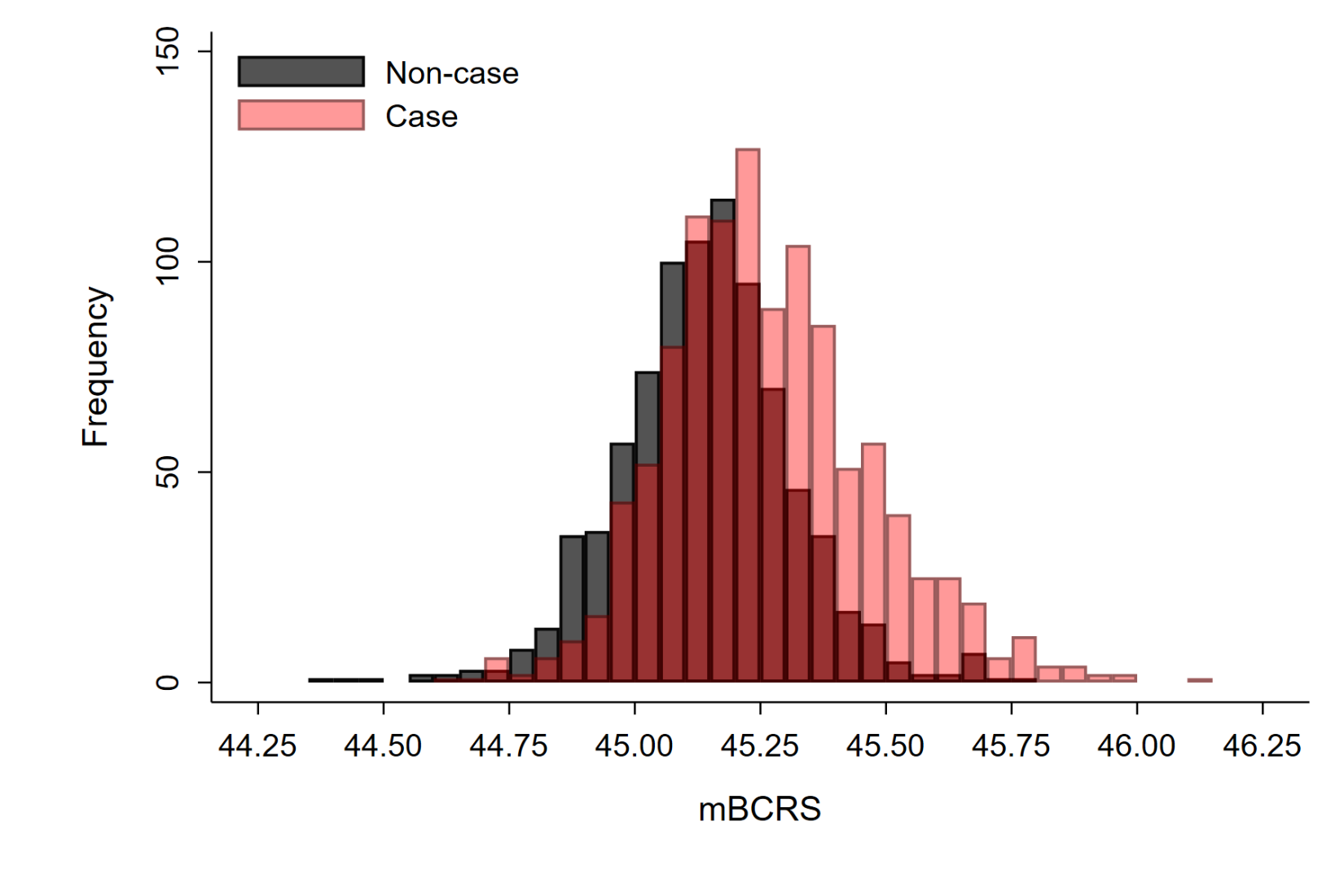


Supplemental Figure 2. Histogram depicting the distribution of mBCRS values in the training set by case status. mBCRS values ranged from 44.40 to 46.14, with a mean of 45.21 (SD=0.20). Non-cases had a mean mBCRS of 45.14 (SD=0.17); cases had a mean mBCRS of 45.27 (SD=0.21).


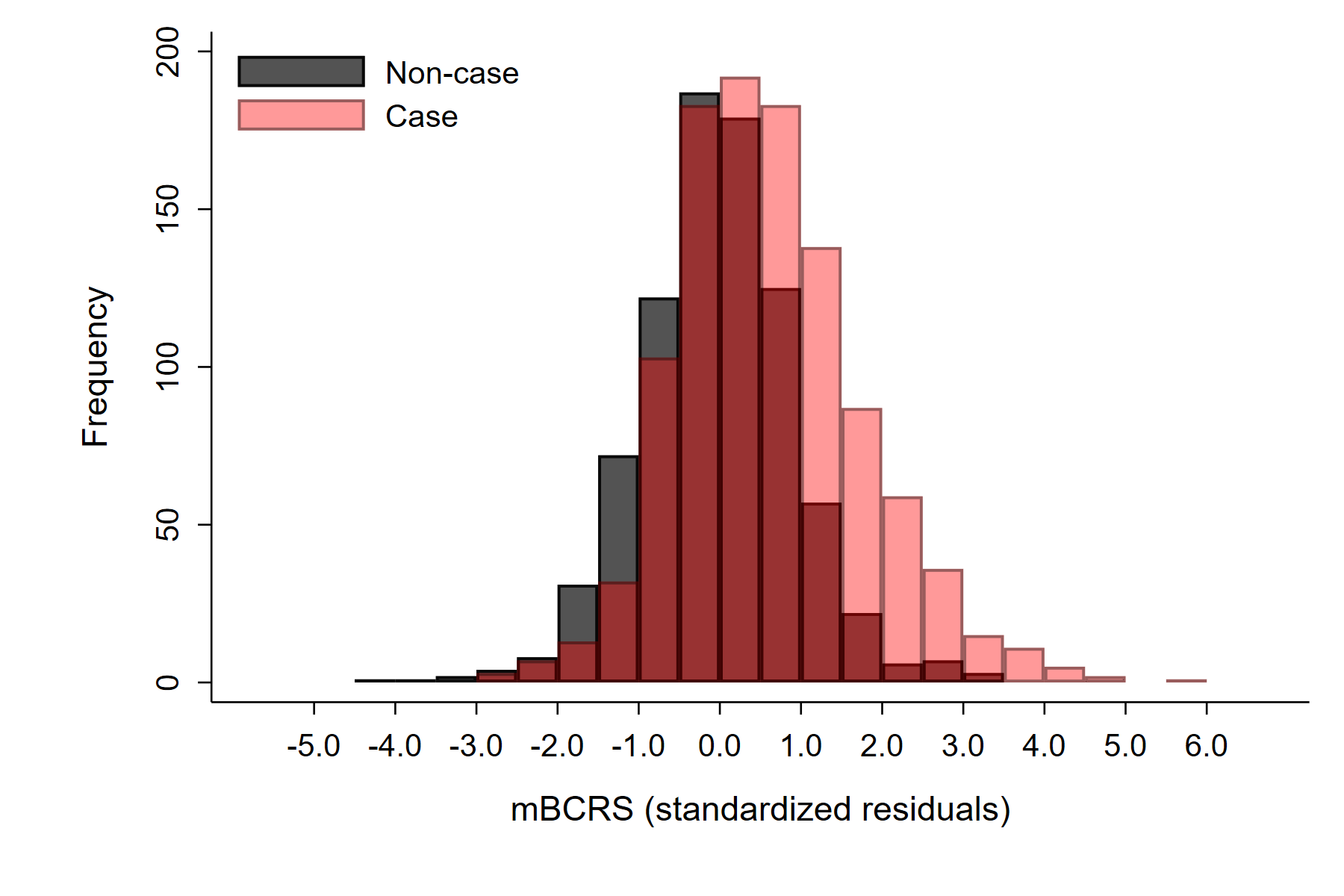


Supplemental Figure 3. Histogram depicting the distribution of the standardized residuals for mBCRS in the training set by case status. mBCRS standardizes residuals ranged from -4.03 to 5.54, with a mean of 0.33 (SD=1.12). Non-cases had a standardized residual mean of -0.04 (SD=0.95). Cases had a standardized residual mean of 0.62 (SD=1.16).


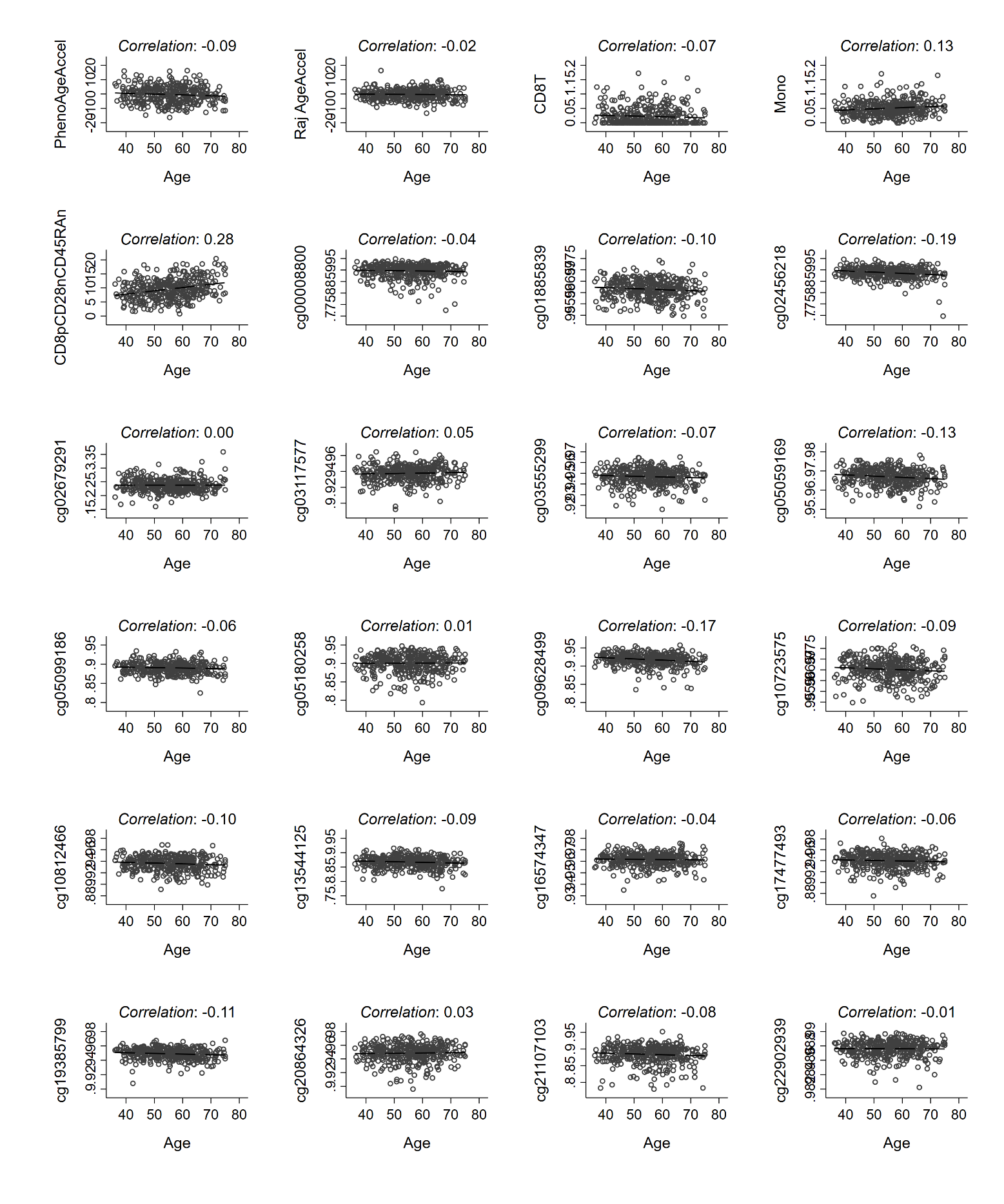


Supplemental Figure 4. mBCRS component correlations with age. Among women sampled as part of the random subcohort and selected in the testing set (N=375), scatter plots, linear fit lines and Pearson correlations shown separately for the 24 mBCRS components and age. Generally, the mBCRS components were individually weakly correlated with age. The strongest positive correlation was for the CD8+CD28-CD45RA- cell type (r= 0.28) and the strongest negative correlation was for cg02456218 (r= -0.19).


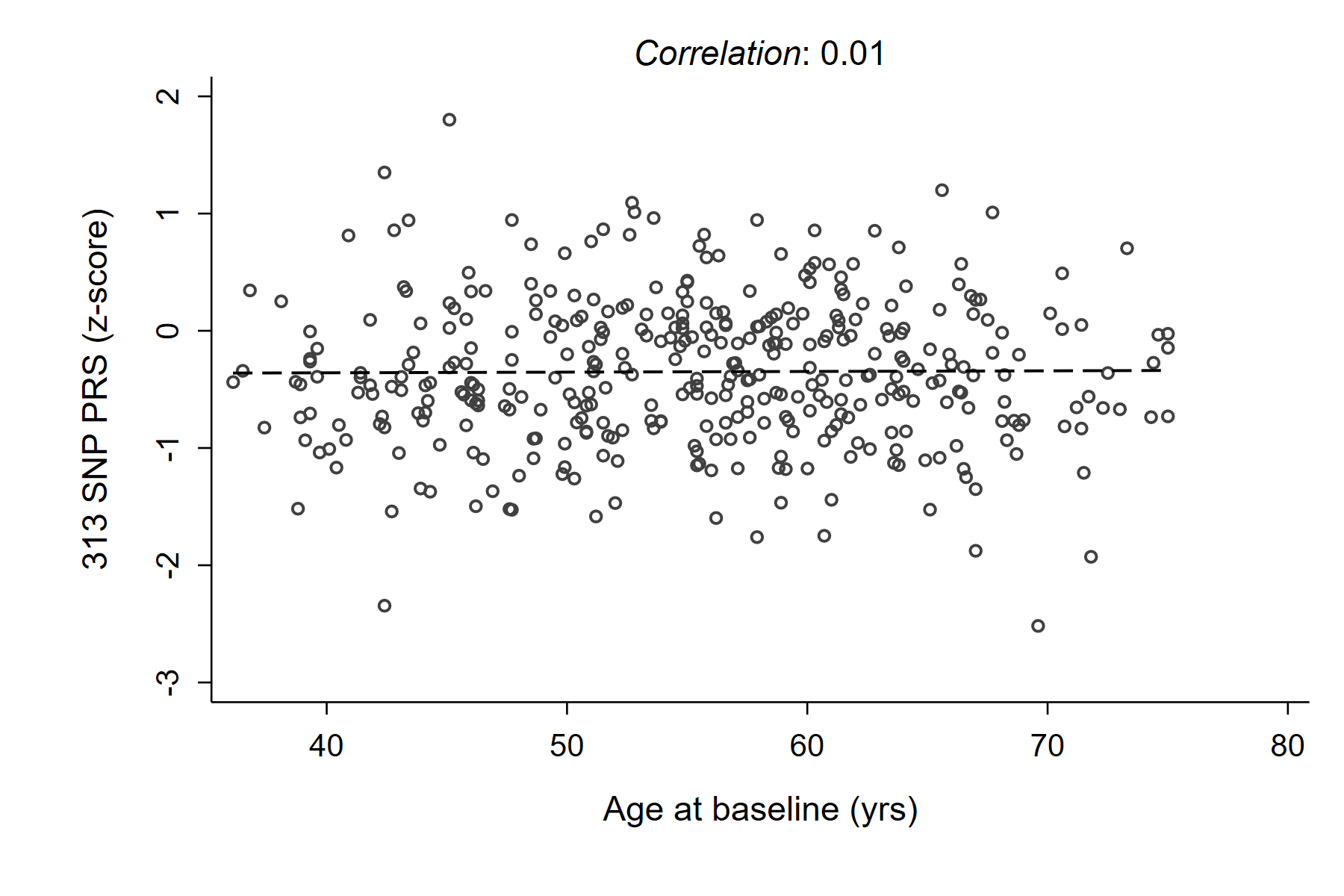


Supplemental Figure 5. Correlation between age and PRS values. Among women sampled as part of the random subcohort and selected into the testing set (n= 375), scatter plot and fit line for the relationship between the PRS and age (Pearson correlation= 0.01).


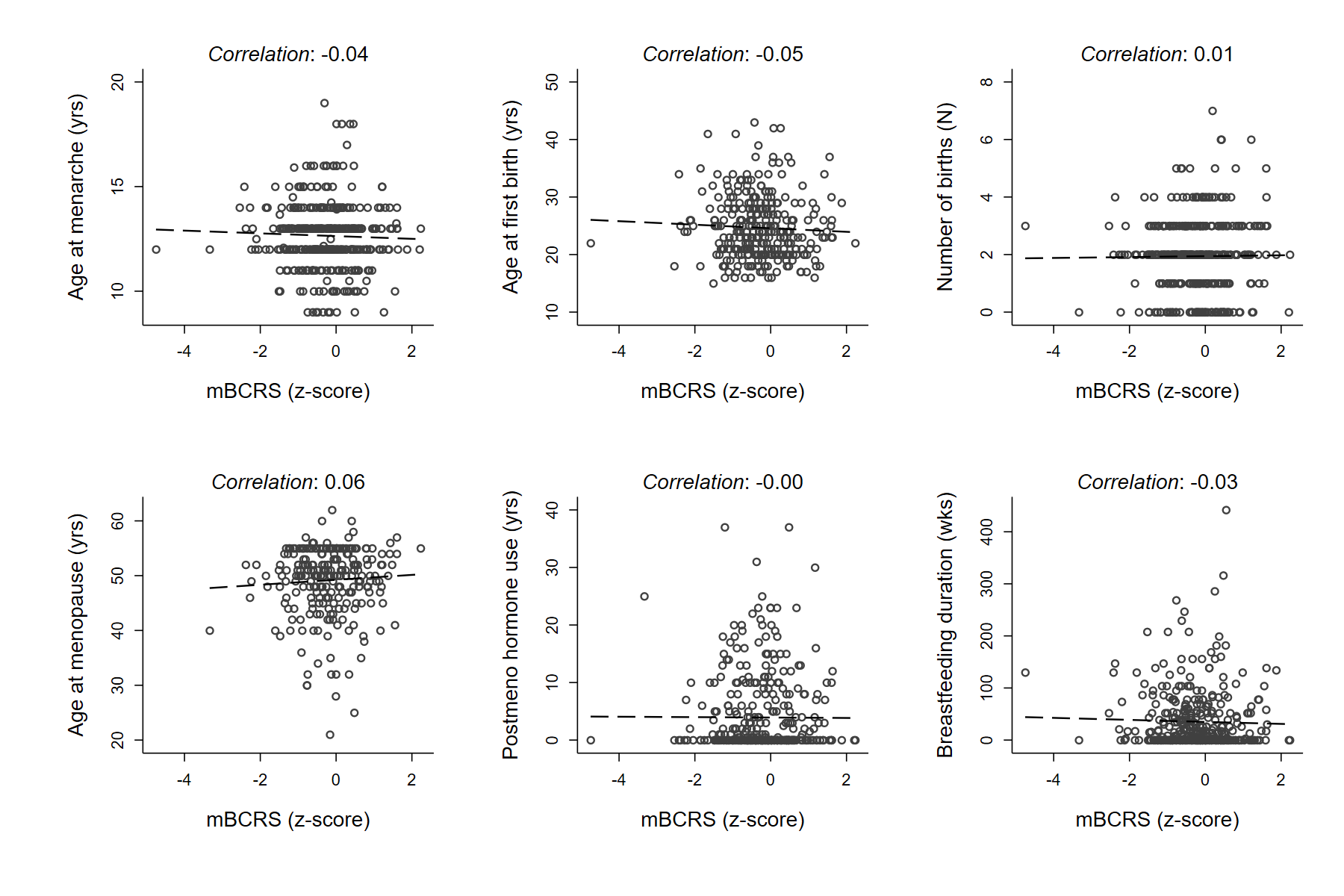


Supplemental Figure 6. mBCRS correlations with selected reproductive factors. Among women sampled as part of the random subcohort and selected into the testing set (n=375), scatter plots, linear fit lines and Pearson correlations for the mBCRS with reproductive factors, including: age at menarche, age at first birth (n= 306) and number of births (top row) and for age at menopause (n= 254), postmenopausal hormone use duration, and breastfeeding duration (bottom row).


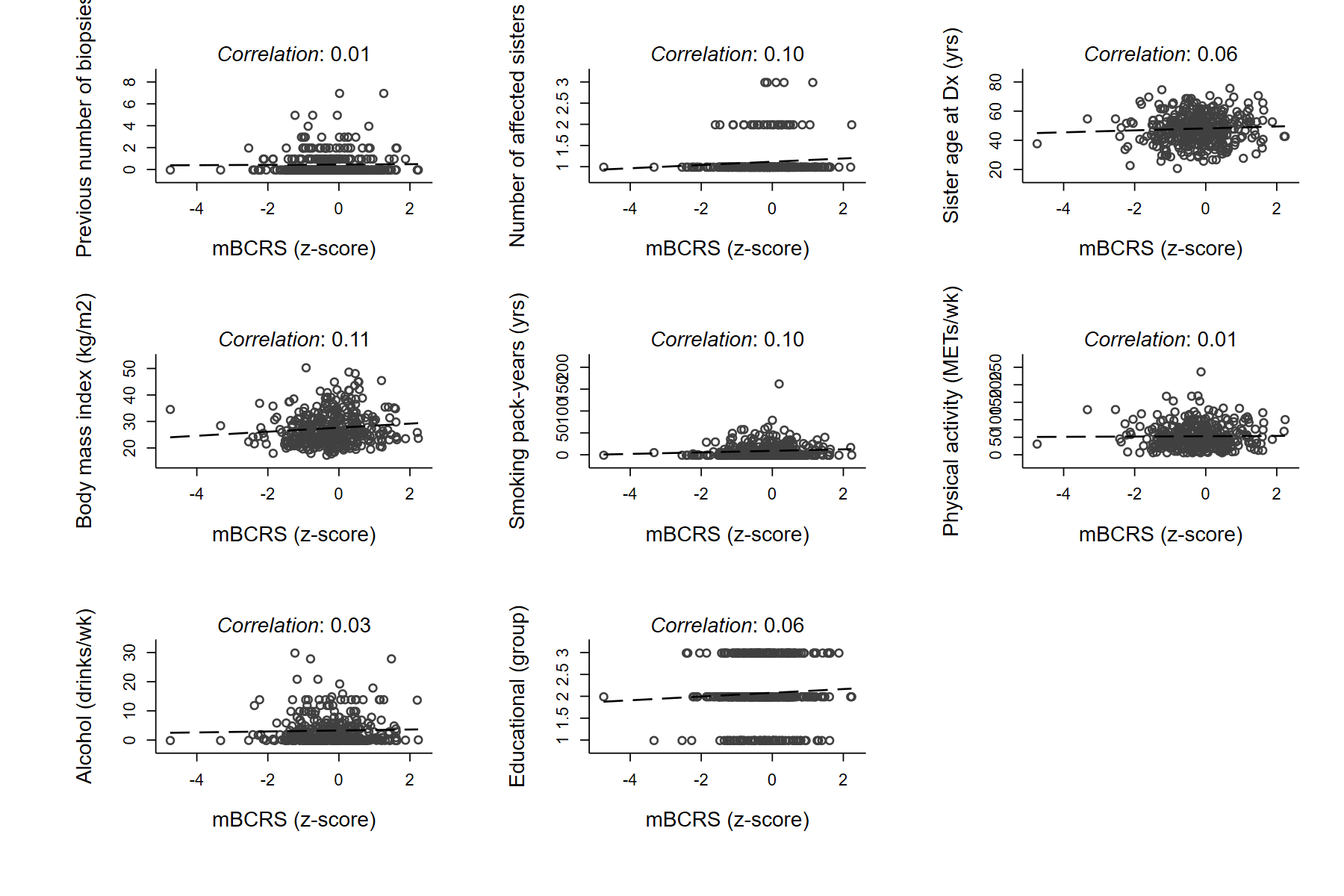


Supplemental Figure 7. mBCRS correlations with selected breast cancer risk factors. Among women sampled as part of the random subcohort and selected into the testing set (n=375), scatter plots, linear fit lines and Pearson correlations for mBCRS and additional breast cancer risk factors including: previous number of breast biopsies, number of sisters diagnosed with breast cancer, and youngest sister proband age at diagnosis (top row), body mass index, smoking history, physical activity (middle row) and alcohol use in the past year, and educational attainment (bottom row).

| Supplemental Table 5. mBCRS tertile associations with breast cancer incidence in the Sister Study internal validation set and the EPIC-Italy external validation set. | | | | | | | | | | |
| --- | --- | --- | --- | --- | --- | --- | --- | --- | --- | --- |
|  | Sister Study testing set | | | |  | | EPIC-Italy sample | | | |
|  | HR (95% CI) | | P-value |  | | OR (95% CI) | | P-value | |  |
| mBCRS percentile |  | |  |  | |  | |  | |  |
| 1-33 | Ref. | |  |  | | Ref. | |  | |  |
| 34-66 | 1.24 (0.88, 1.76) | | 0.22 |  | | 2.94 (1.67, 5.20) | | 2.0×10^-4^ | |  |
| 67-100 | 2.54 (1.78, 3.63) | | 2.9×10^-7^ |  | | 4.18 (2.33, 7.49) | | 1.7×10^-6^ | |  |
| P-trend |  | 3.6×10^-7^ | | |  | |  | | 1.8×10^-6^ | |
| Abbreviations: hazard ratio, HR; odds ratio, OR  Hazard ratios were calculated using Cox regression models in the Sister Study testing set, with age treated as the time-scale; Odds ratios were calculated using unconditional logistic regression in the EPIC-Italy sample, model included age at blood draw | | | | | | | | | | |


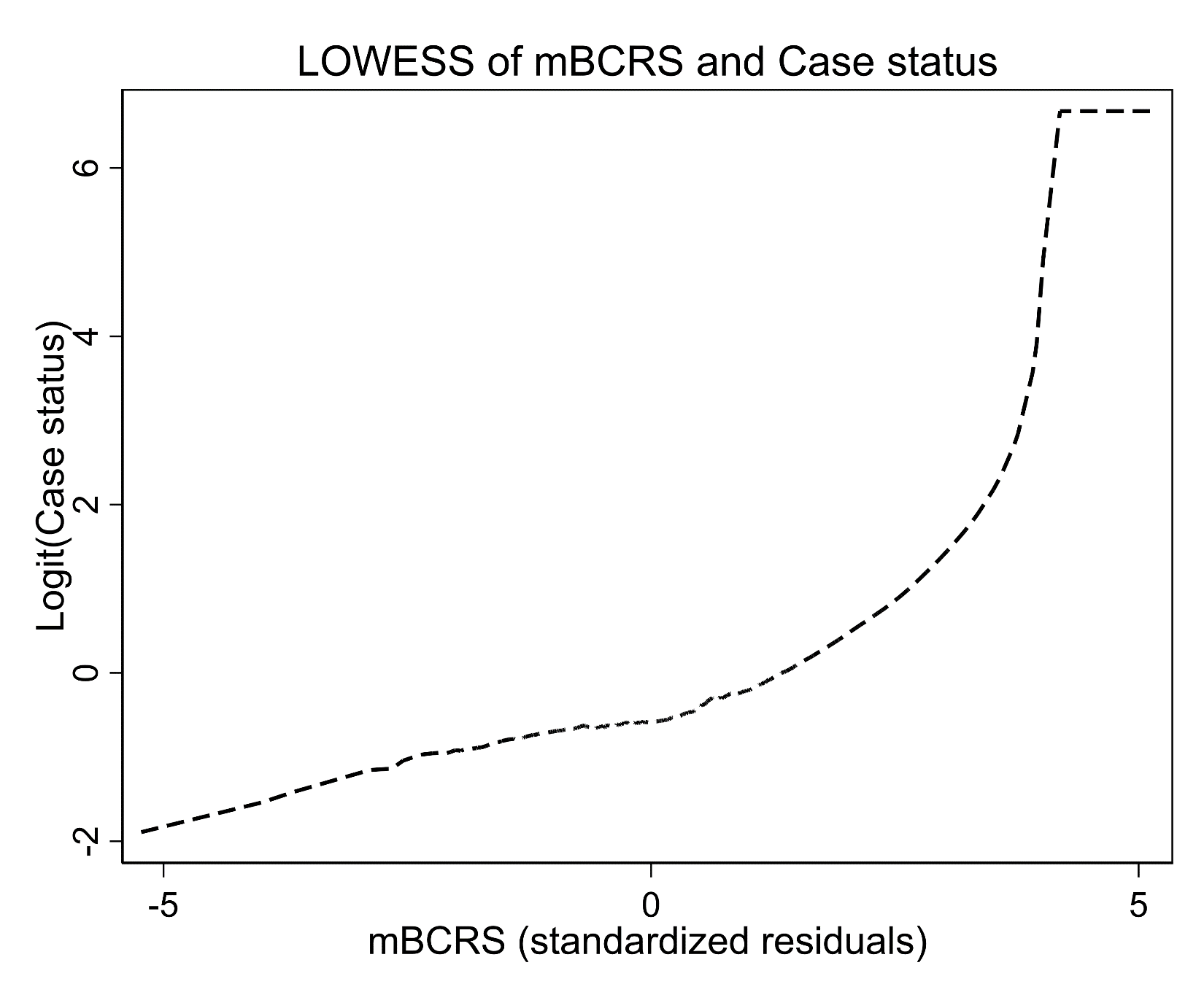


Supplemental Figure 8. LOWESS plot of mBCRS by case status. Among the Sister Study testing set, a LOWESS smoother with a bandwidth of 0.75 shows evidence of non-linearity. The association between mBCRS and breast cancer risk is stronger among women with the highest mBCRS values.


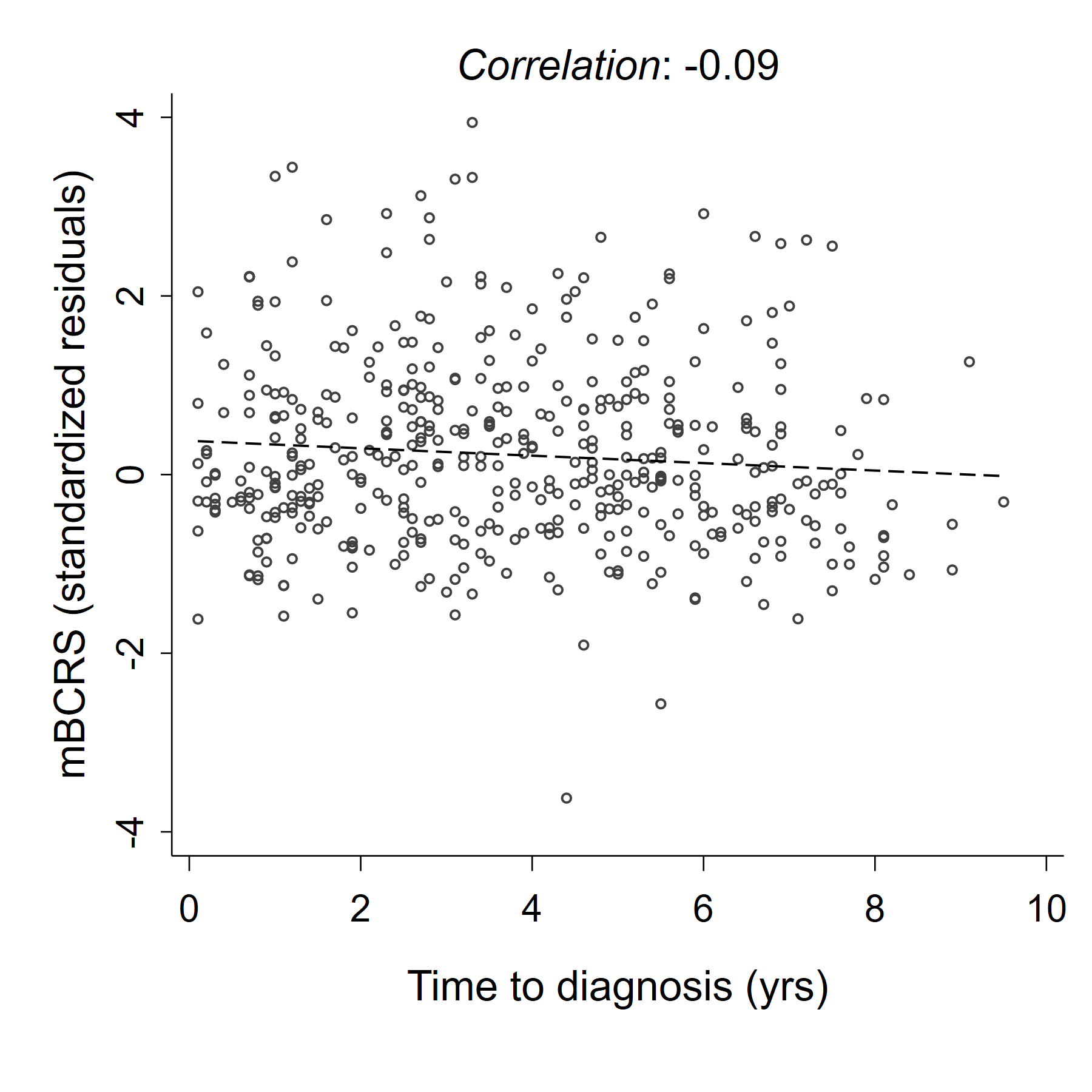


Supplemental Figure 9. Correlation between mBCRS and time to diagnosis among cases. Among women diagnosed with breast cancer in the testing set (n= 443), scatter plot and linear fit line for the relationship between mBCRS score and time to diagnosis. The Pearson correlation for mBCRS and time to diagnosis was -0.09 (p= 0.05).

| Supplemental Table 6. mBCRS associations (per covariate-adjusted SD) with breast cancer incidence in the Sister Study testing set, stratified by age at blood draw, body mass index, menopause status and proband age at diagnosis. | | | | | | | | | | |
| --- | --- | --- | --- | --- | --- | --- | --- | --- | --- | --- |
| Age at blood draw | ≤ 50 | |  | 51 – 65 | | |  | > 65 | | P-interaction |
|  | HR (95% CI)^1^ | P-value |  | HR (95% CI)^1^ | P-value |  | | HR (95% CI)^1^ | P-value |  |
| mBCRS | 1.61 (1.17, 2.21) | 3.5×10^-3^ |  | 1.87 (1.48, 2.36) | 1.1×10^-7^ |  | | 1.74 (1.23, 2.47) | 1.6×10^-3^ | 0.73 |
| Body mass index | < 25 | |  | 25 – 30 | |  | | > 30 |  | P-interaction |
|  | HR (95% CI)^1^ | P-value |  | HR (95% CI)^1^ | P-value |  | | HR (95% CI)^1^ | P-value |  |
| mBCRS | 1.79 (1.40, 2.30) | 3.7×10^-6^ |  | 2.17 (1.59, 2.98) | 1.3×10^-6^ |  | | 1.59 (1.12, 2.25) | 9.4×10^-3^ | 0.52 |
| Menopause status | Premenopausal | |  | Postmenopausal | |  | |  | | P-interaction |
|  | HR (95% CI)^1^ | P-value |  | HR (95% CI)^1^ | P-value |  | |  |  |  |
| mBCRS | 1.79 (1.36, 2.35) | 3.4×10^-5^ |  | 1.77 (1.44, 2.18) | 8.8×10^-8^ |  | |  |  | 0.93 |
| Proband age at diagnosis | < 50 | |  | ≥ 50 | |  | |  |  | P-interaction |
|  | HR (95% CI)^1^ | P-value |  | HR (95% CI)^1^ | P-value |  | |  |  |  |
| mBCRS | 2.00 (1.57, 2.54) | 1.5×10^-8^ |  | 1.65 (1.29, 2.10) | 5.0×10^-5^ |  | |  |  | 0.28 |
| ^1^Per covariate-adjusted standard deviation increase in mBCRS score  Sample sizes for age at blood draw: ≤ 50 n=186 with 80 events; 51 – 65 n=475 with 267 events; 65+ n= 151 with 96 events  Sample sizes for body mass index: < 25 n=322 with 174 events; 25 – 30 n=231 with 131 events; > 30 n= 241 with 138 events  Sample sizes for menopause status: premenopausal n=238 with 117 events; postmenopausal n=556 with 326 events  Sample sizes for proband age at diagnosis: ≤ 50 n=454 with 244 events; > 50 n=340 with 199 events  Breast cancer covariates include: age, menopause status, body mass index, interaction-term for BMI and menopause, physical activity, alcohol consumption, age at first birth (among parous), total number of births, age at menarche, menopause age (among postmenopausal), smoking pack years, previous number of breast biopsies, family history of breast cancer (number of affected sisters, youngest age of sister’s diagnosis) and durations of postmenopausal hormone use and breastfeeding. | | | | | | | | | |  |

| Supplemental Table 7. mBCRS associations (per covariate-adjusted SD) with breast cancer incidence by invasiveness and estrogen receptor status, and tests for etiologic heterogeneity in the Sister Study testing set | | | | | | | | | | |  |
| --- | --- | --- | --- | --- | --- | --- | --- | --- | --- | --- | --- |
|  | Among all breast cancers | | | | | | |  | Invasive vs DCIS^2^ |  |  |
|  | Invasive | | |  | Ductal carcinoma *in situ* | | |  |  |  |  |
|  | HR (95% CI)^1^ | Z-score | P-value |  | HR (95% CI)^1^ | Z-score | P-value |  | OR (95% CI) | P-het^3^ |  |
| mBCRS | 1.90 (1.58, 2.29) | 6.75 | 1.5×10^-11^ |  | 1.50 (1.20, 1.89) | 3.53 | 4.1×10^-4^ |  | 1.20 (1.00, 1.45) | 0.05 |  |
|  | Among invasive breast cancers | | | | | | |  | ER-positive vs ER-negative^2^ |  | |
|  | Estrogen receptor positive | | |  | Estrogen receptor negative | | |  |  |  |  |
|  | HR (95% CI)^1^ | Z-score | P-value |  | HR (95% CI)^1^ | Z-score | P-value |  | OR (95% CI) | P-het^3^ |  |
| mBCRS | 1.92 (1.58, 2.33) | 6.55 | 5.9×10^-11^ |  | 1.71 (1.18, 2.47) | 2.84 | 4.6×10^-3^ |  | 1.10 (0.84, 1.44) | 0.47 |  |
| ^1^Per covariate-adjusted standard deviation increase in marker score. Sample sizes for Cox regression modes: invasive n= 682 with 326 events; DCIS n= 487 with 117 events; ER-positive n= 636 with 282 events; ER-negative n= 410 with 43 events  ^2^Case-only analysis for etiologic heterogeneity comparing invasive vs DCIS (sample n= 443; 117 DCIS, 326 invasive) or invasive estrogen receptor (ER) positive vs invasive ER negative (sample n= 325, 282 ER-positive, 43 ER-negative, 1 missing ER status).  ^3^P-heterogeneity calculated using case-only analysis for etiologic heterogeneity  Breast cancer risk factors include: age, menopause status, body mass index, interaction-term for BMI and menopause, physical activity, alcohol consumption, age at first birth (among parous), total number of births, age at menarche, menopause age (among postmenopausal), smoking pack years, previous number of breast biopsies, family history of breast cancer (number of affected sisters, youngest age of sister’s diagnosis) and durations of postmenopausal hormone use and breastfeeding. | | | | | | | | | | |  |

| Supplemental Table 8. Multivariate OPERA (95% CI) estimates of odds ratios per adjusted standard deviation from separate and combined analysis of mBCRS component classes (DNAm estimators & individual CpGs), adjusted for breast cancer risk factors and standardized | | | |
| --- | --- | --- | --- |
|  | DNAm estimators only | Individual  CpGs only | DNAm estimators + CpGs combined |
| PhenoAgeAccel | 1.04 (0.87, 1.23) | ----- | 1.08 (0.90, 1.29) |
| Raj AgeAccel | 1.14 (0.96, 1.35) | ----- | 1.07 (0.89, 1.29) |
| CD8+ T-cells | 0.95 (0.82, 1.11) | ----- | 0.94 (0.79, 1.12) |
| Monocytes | 0.97 (0.83, 1.12) | ----- | 0.95 (0.81, 1.11) |
| CD8+CD28-CD45RA- | **0.79 (0.68, 0.93)** | ----- | **0.79 (0.67, 0.93)** |
| cg00008800 | ----- | 1.00 (0.85, 1.18) | 1.04 (0.88, 1.22) |
| cg01885839 | ----- | 1.02 (0.88, 1.17) | 1.01 (0.87, 1.16) |
| cg02456218 | ----- | 0.92 (0.79, 1.08) | 0.93 (0.79, 1.09) |
| cg02679291 | ----- | 1.12 (0.95, 1.32) | 1.13 (0.96, 1.34) |
| cg03117577 | ----- | **1.21 (1.03, 1.42)** | **1.18 (1.00, 1.39)** |
| cg03555299 | ----- | 1.00 (0.84, 1.19) | 1.00 (0.85, 1.19) |
| cg05059169 | ----- | 1.13 (0.97, 1.32) | 1.12 (0.96, 1.31) |
| cg05099186 | ----- | 1.03 (0.87, 1.21) | 1.02 (0.86, 1.20) |
| cg05180258 | ----- | 0.97 (0.83, 1.14) | 0.98 (0.84, 1.15) |
| cg09628499 | ----- | 0.98 (0.74, 1.15) | 0.97 (0.83, 1.15) |
| cg10723575 | ----- | 0.89 (0.77, 1.04) | 0.90 (0.77, 1.05) |
| cg10812466 | ----- | 1.05 (0.89, 1.24) | 1.02 (0.87, 1.21) |
| cg13544125 | ----- | 1.06 (0.91, 1.24) | 1.05 (0.90, 1.23) |
| cg16574347 | ----- | 1.02 (0.86, 1.20) | 1.02 (0.86, 1.20) |
| cg17477493 | ----- | 0.94 (0.80, 1.09) | 0.92 (0.79, 1.08) |
| cg19385799 | ----- | **0.86 (0.75, 1.00)** | **0.86 (0.74, 0.99)** |
| cg20864326 | ----- | 0.88 (0.75, 1.04) | 0.86 (0.73, 1.02) |
| cg21107103 | ----- | 0.91 (0.78, 1.06) | 0.90 (0.77, 1.05) |
| cg22902939 | ----- | **1.46 (1.24, 1.72)** | **1.47 (1.24, 1.74)** |
| Breast cancer risk factors include: age, menopause status, body mass index, interaction-term for BMI and menopause, physical activity, alcohol consumption, age at first birth (among parous), total number of births, age at menarche, menopause age (among postmenopausal), smoking pack years, previous number of breast biopsies, family history of breast cancer (number of affected sisters, youngest age of sister’s diagnosis), educational attainment, and durations of postmenopausal hormone use and breastfeeding. | | | |

**References**

1. Horvath S. DNA methylation age of human tissues and cell types. *Genome Biol* 2013;14(10):R115.

2. Hannum G, Guinney J, Zhao L, et al. Genome-wide methylation profiles reveal quantitative views of human aging rates. *Mol Cell* 2013;49(2):359-67.

3. Levine ME, Lu AT, Quach A, et al. An epigenetic biomarker of aging for lifespan and healthspan. *Aging (Albany NY)* 2018;10(4):573-91.

4. Horvath S, Oshima J, Martin GM, et al. Epigenetic clock for skin and blood cells applied to Hutchinson Gilford Progeria Syndrome and ex vivo studies. *Aging (Albany NY)* 2018;10(7):1758-75.

5. Lu AT, Quach A, Wilson JG, et al. DNA methylation GrimAge strongly predicts lifespan and healthspan. *Aging (Albany NY)* 2019;11(2):303-27.

6. Chen BH, Marioni RE, Colicino E, et al. DNA methylation-based measures of biological age: meta-analysis predicting time to death. *Aging (Albany NY)* 2016;8(9):1844-65.

7. Lu AT, Seeboth A, Tsai PC, et al. DNA methylation-based estimator of telomere length. *Aging (Albany NY)* 2019;11(16):5895-923.

8. Houseman EA, Accomando WP, Koestler DC, et al. DNA methylation arrays as surrogate measures of cell mixture distribution. *BMC Bioinformatics* 2012;13:86.

9. McCartney DL, Hillary RF, Stevenson AJ, et al. Epigenetic prediction of complex traits and death. *Genome Biol* 2018;19(1):136.

10. Xu Z, Sandler DP, Taylor JA. Blood DNA methylation and breast cancer: A prospective case-cohort analysis in the Sister Study. *J Natl Cancer Inst* 2019.
